# 7-Dialkylaminocoumarin Oximates: Small Molecule Fluorescent “Turn-On” Chemosensors for Low-Level Water Content in Aprotic Organic Solvents

**DOI:** 10.3390/molecules22081340

**Published:** 2017-08-12

**Authors:** Marek Cigáň, Miroslav Horváth, Juraj Filo, Klaudia Jakusová, Jana Donovalová, Vladimír Garaj, Anton Gáplovský

**Affiliations:** 1 Faculty of Natural Sciences, Institute of Chemistry, Comenius University, Ilkovičova 6, Mlynská dolina CH-2, SK-842 15 Bratislava, Slovakia; mirek.horvath@gmail.com (M.H.); filo@fns.uniba.sk (J.F.); jakusova@fns.uniba.sk (K.J.); donovalova@fns.uniba.sk (J.D.); gaplovsky@fns.uniba.sk (A.G.); 2 Department of Pharmaceutical Chemistry, Faculty of Pharmacy, Comenius University, Odbojárov 10, SK-832 32 Bratislava, Slovakia; garajv@fpharm.uniba.sk

**Keywords:** water sensing, fluorescent probe, coumarin, oxime/oximate equilibrium, dark excited state, photoinduced electron transfer

## Abstract

The water sensing properties of two efficient two-component fluorescent “turn-on” chemo-sensors based on the 7-dialkylaminocoumarin oxime acid-base equilibrium were investigated. Interestingly, although simple frontier orbital analysis predicts an intramolecular photoinduced electron transfer quenching pathway in conjugated oximates, TD-DFT (Time-dependent density functional theory) quantum chemical calculations support non-radiative dark S_1_ excited state deactivation as a fluorescence quenching mechanism. Due to the acid-base sensing mechanism and sensitive “turn-on” fluorescent response, both studied coumarin aldoxime chemosensors exhibit rapid response to low-level water content in polar aprotic solvents, with detection limits comparable to chemodosimeters or chemosensors based on interpolymer π-stacking aggregation.

## 1. Introduction

A qualitative and quantitative detection of low-level water present as an impurity in organic solvents has great significance in several fields of chemistry and industry processes (pharmaceutical manufacturing, food processing, production of anhydrous solvents and chemical reagents, petroleum fuel industry, paper production and biomedical or environmental monitoring) [1,2,3,4,5,6,7,8,9,10,11,12,13,14,15,16,17,18,19,20,21,22,23]. Many analytical approaches and techniques have been established for the determination of water in a wide variety of organic solvents, such as Karl Fischer titration and gas chromatography methods on the laboratory scale, and electrochemical/electrophysical methods particularly for large-scale industry processes. However, electrical sensors suffer often from the lack of insufficient portability/precision and exhibit undesirable sensitivity to electromagnetic interference [4]. Laboratory methods have the disadvantage of requiring time-consuming sample preparation, inability of real-time monitoring of the water content, a need for skilled personnel and special equipment and interference from other co-existing species [1,2,3,4,5,6,7,8,9,10,11,12,13,14,15,16,17,18,19,20,21,22,23]. Moreover, the Karl–Fisher titration is less sensitive in aprotic and non-alcoholic solvents [19]. Therefore, the development of optical (colorimetric and fluorescent) sensors for water sensing have drawn considerable attention due to their flexibility in readout, the possibility of remote and in situ monitoring, as well as their easy and low-cost fabrication [3,4].

In particular, low-cost, rapid, simple and sensitive fluorescence methods, including photo-induced electron transfer (PET), intramolecular charge transfer (ICT), excited state intramolecular proton transfer (ESIPT), Förster resonance energy transfer (FRET), aggregation induced emission (AIE), aggregation based monomer-excimer/exciplex switching, interpolymer π-stacking aggregation, competitive ligand/acid-base sensing mechanism, have been widely investigated for this purpose. An excellent review related to recent advances in the field of colorimetric and fluorescent sensors for low-level water content, categorized by sensing mechanism, was recently published by Kim et al. [24].

Our research group also recently published the sensing properties of four donor/acceptor *para*- substituted 7-dimethylaminocoumarin (7-dialkylamino-2*H*-chromen-2-one) phenylsemicarbazones for low-level water content in aprotic polar organic solvents [25]. Competition with water in a reversible acid-base reaction led to effective two-component coumarin phenylsemicarbazone/anion optical chemosensors. Despite the fact their sensing mechanism is based on an acid-base equilibrium, they can be classified as “competitive ligand-based” chemosensors, similar to the quinoline or phenolic dyes published by Kim et al. and Moon et al., respectively, with detection limits of 0.16–0.17 wt % [10,26]. Determined detection limits (LOD) for water by the studied phenylsemicarbazones were amongst the lowest detection limits published in the literature and they can compete in sensitivity with chemodosimeters (LOD: 0.0016–0.0026 wt %) [25].

**Scheme 1:**
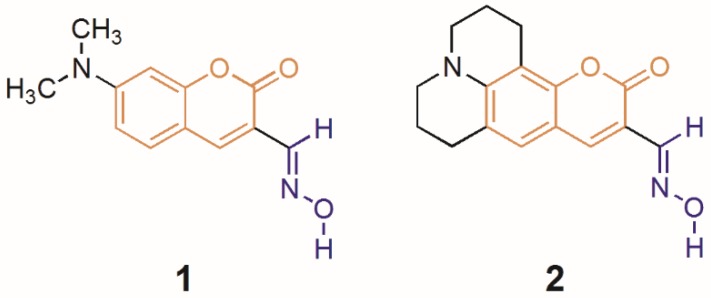
Molecular structures of the studied coumarin oximes **1** and **2**.

Herein, we report results of a comprehensive investigation of the water sensing properties and sensing/response mechanism of two similar two-component fluorescent “turn-on” chemosensors based on the 7-dialkylaminocoumarin oxime acid-base equilibrium (Scheme 1). 7-Dialkylaminocoumarin oxime chemosensors exhibit similar detection limits as the corresponding coumarin phenyl- semicarbazones and, contrary to phenylsemicarbazones, their fluorescence is not quenched by basic CH_3_COO^−^ anions. In general, 7-dialkylaminocoumarins are arguably the most important and applicable subset of coumarin fluorescent probes due to their strong fluorescent response dependence on polarity, hydrogen bonding ability, pH, presence of quest anions, various metal ions, biologically important compounds, chemical warfare agents and micro-viscosity or rotational hindrance in their local environment [11,27,28,29,30,31,32,33,34,35]. Moreover, 7-donor-subsituted coumarin oximes/oximates are known fluorescent probes for HOCl, chloramine and chemical warfare simulants/organophosphorus nerve agents [36,37,38,39].

## 2. Results and Discussion

### 2.1. Spectral Characteristics

Both studied 7-dialkylaminocoumarin oximes **1** and **2** exhibit strong blue-violet light absorption and high fluorescent quantum yields (Φ_F_ > 0.8) with almost constant 3–4 ns excited state lifetimes in all three polar aprotic solvents used (Table 1). High Φ_F_ and a significant bathochromic shift of both the absorption and fluorescence maxima (λ_A_ and λ_F_) compared to the parent 2-oxo-2*H*-chromene clearly support a characteristic charge-transfer character of the dialkylaminocoumarin excited state with only a small portion of competing non-radiative deexcitation pathways (intersystem crossing and/or intramolecular rotation; Table 1—τ_1_) [40]. Whereas the oxime **1** shows lower light absorption intensity (ε_A_) and its λ_A_ and λ_F_ are hypsochromically shifted compared to the previously studied acceptor 7-dimethylaminocoumarin phenylsemicarbazones (~15 nm), both oximes exhibit higher emission efficiency, particularly in MeCN [25]. As expected, due to the restricted intramolecular rotation of the dialkylamino group, the oxime **2** Φ_F_ reaches almost 100% in highly polar DMF and DMSO.

### 2.2. Water Sensing

Although fluoride (F^−^) anion addition to oxime **1** and **2** solutions practically does not affect their light absorption and leads only to small hypsochromic shift of λ_A_ and non-significant decrease in extinction intensity (Supplementary Materials ESI Figures S1–S4), solution fluorescence almost completely disappears (ESI Figures S5 and S6). Practically unchanged fluorescence lifetime τ_2_ and significant short-lifetime τ_1_ component contribution in fluorescence decay curve after F^−^ addition indicate a chemical reaction (static quenching) between oxime and F^−^ and simultaneously exclude dynamic collisional quenching of oxime fluorescence by F^−^ anion (ESI Figure S7; collisional process should lead to gradual τ_2_ decrease with increasing F^−^ content). Unfortunately, changes in emission characteristics are not sufficiently sensitive to allow studied coumarin oximes to achieve the status of fluorescent probes for this anion.

**Figure 1:**
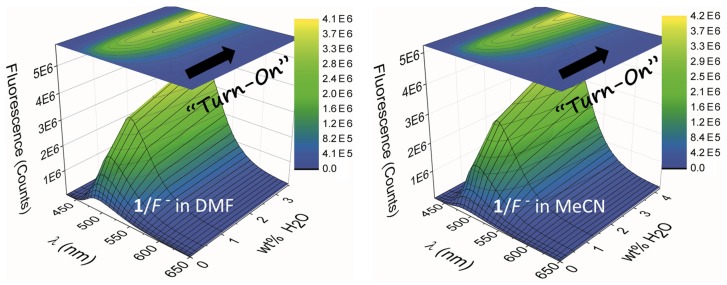
Evolution of the fluorescence spectrum of two-component coumarin oxime **1**/F^−^ sensor in DMF and MeCN during its titration with water (DMF: 2 × 10^−5^ mol L^−1^ **1** + 1 × 10^−2^ mol L^−1^ TBA^+^F^−^; MeCN: 5 × 10^−5^ mol L^−1^ **1** + 1 × 10^−2^ mol L^−1^ TBA^+^F^−^; λ_EX_ = λ_A(oxime)_; *T* = 298.15 K).

**Figure 2:**
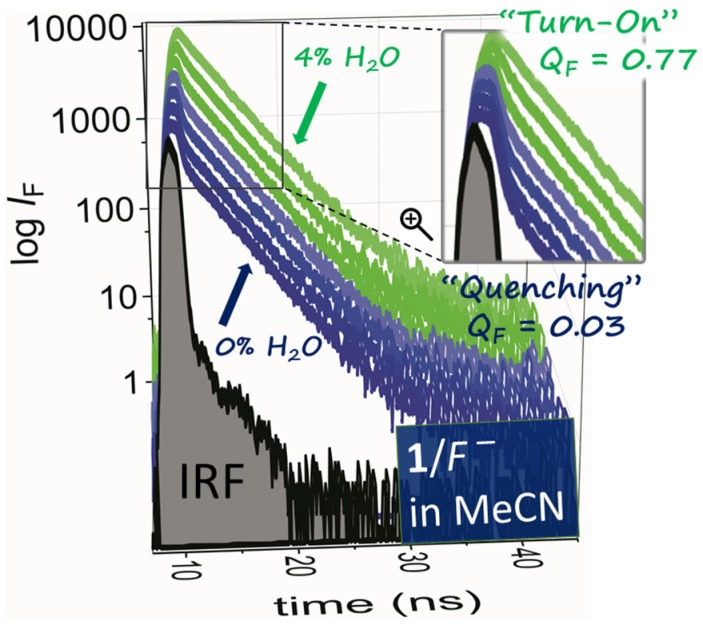
Evolution of the fluorescence lifetime of two-component coumarin oxime **1**/ F^−^ sensor in MeCN during its titration with water (5 × 10^−5^ mol L^−1^ **1** + 1 × 10^−2^ mol L^−1^ TBA^+^F^−^; *T* = 298.15 K; λ_EX_ = λ_A(oxime)_ = 413 nm ; IRF—instrument response function; results of fitting: Initial state (F^−^ addition): τ_1_ = 0.2 ns (36%), τ_2_ = 3.1 ns (64%), χ^2^ = 1.192; Water addition (4% (*v/v*)): τ_1_ = 1.0 ns (6%), τ_2_ = 3.3 ns (94%), χ^2^ = 1.130).

However, already traces of water (gradual low-level water addition) result in initial light absorption and intense fluorescence recovery (Figure 1 and ESI Figures S1–S4). The Φ_F_ practically returns to its initial high value (Φ_F_ ~ 0.8) and the long-lifetime component τ_2_ contributes again dominantly to the overall fluorescence (Figure 2 vs. Table 1). The two-component oxime/F^−^ system therefore could be used as a chemosensor for low-level water content in aprotic organic solvents.

**Figure 3:**
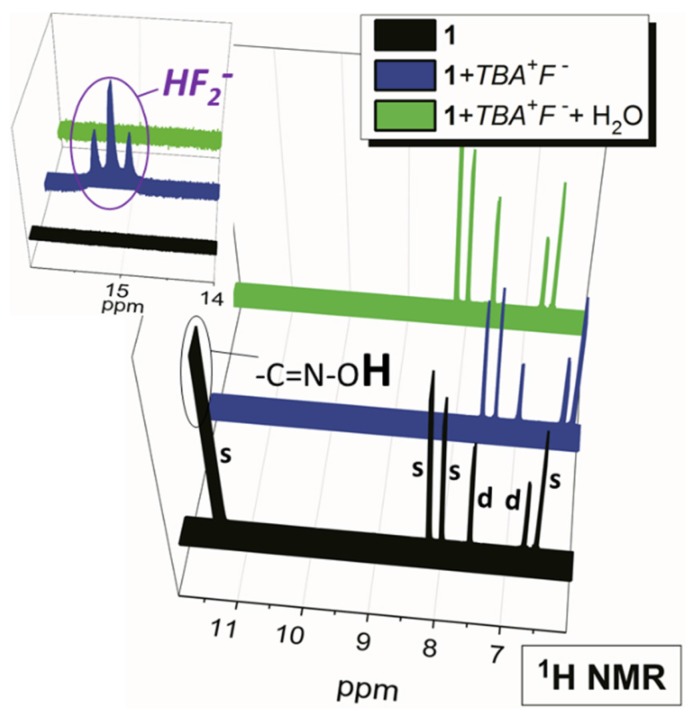
^1^H NMR spectrum of 7-dimethylaminocoumarin oxime **1** in DMSO-*d*_6_ before and after F^−^ anion (TBA^+^F^−^) and subsequent water addition (*c*_1_ = 5 × 10^−4^ mol L^−1^; *c*_F_^−^ = 1 × 10^−1^ mol L^−1^; 4% (*v/v*) of water; *T* = 298.15 K).

As indicated by ^1^H-NMR spectroscopy, the addition of water returns all C-H signals to the initial state (before the F^−^ anion addition; Figure 3 and ESI Figures S8 and S9). Only the =N-O-H proton in ^1^H-NMR spectra of both oximes is not visible due to fast chemical exchange with water. Its initial downfield position (~11 ppm) in pure oxime **1** and **2** solutions indicates the presence of five-membered intramolecular hydrogen bonding (ESI Table S1 and ESI Figure S10—conformers I-III vs. conformers IV and V).

**Scheme 2:**
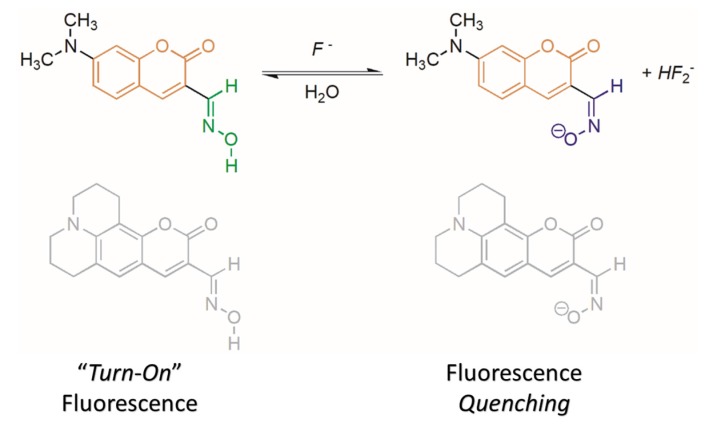
Acid-base sensing mechanism for low-level water content determination in polar aprotic solvents using two-component coumarin oxime/F^−^ chemosensors **1**/F^−^ and **2**/F^−^.

The sensing mechanism therefore includes back aldoxime base (oximate) protonation by water as acid to the corresponding initial oxime after previous most acidic =N-O-H hydrogen deprotonation by the strongly basic F^−^ anion (Scheme 2).

Because the shape of the fluorescence decay curve after F^−^ addition (ESI Figure S7) and oximate fluorescence insensitivity to matrix viscosity/rigidity (ESI Figure S11) exclude dynamic quenching and intramolecular rotation (C=N isomerization or TICT state formation), respectively, as dominant quenching mechanism, the quenching of oxime fluorescence in the presence of F^−^ anions could result from dark-state quenching or reductive photoinduced electron transfer (PET). Already Anslyn et al. [39] indicated PET quenching mechanism in similar 4-butyl-substituted 7-dimethylaminocoumarin oximate anion. However, due to absence of separated donor and acceptor molecule parts by unconjugated bridge and thus isolated acceptor part excitation, we do not assume typical PET in the oximate anion (although simple frontier orbital analysis of artificially separated donor and acceptor parts allows the PET quenching pathway—ESI Table S2; ESI Scheme S1).

**Figure 4:**
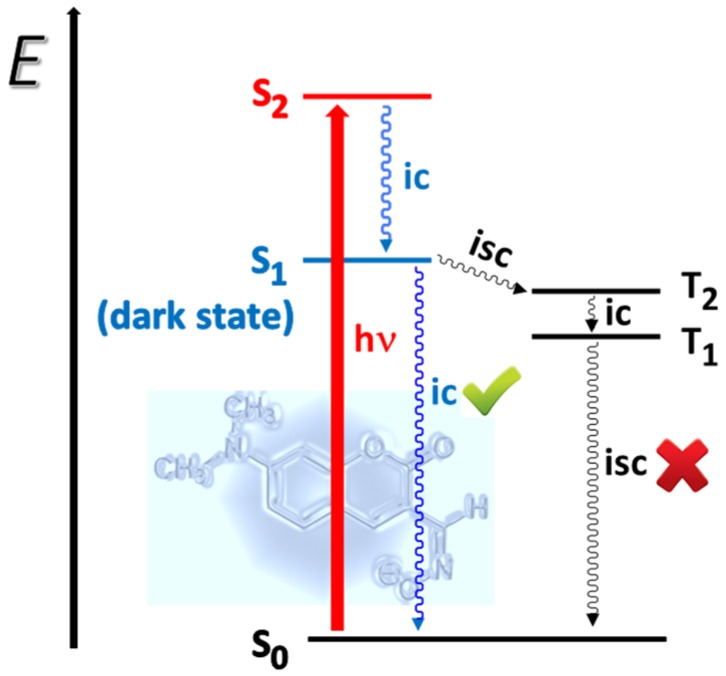
Fluorescence quenching mechanism of 7-dimethylaminocoumarin oxime **1** in the presence of strongly basic F^−^ anions (state diagram and de-excitation pathways for 7-dimethylaminocoumarin oximate anion).

**Figure 5:**
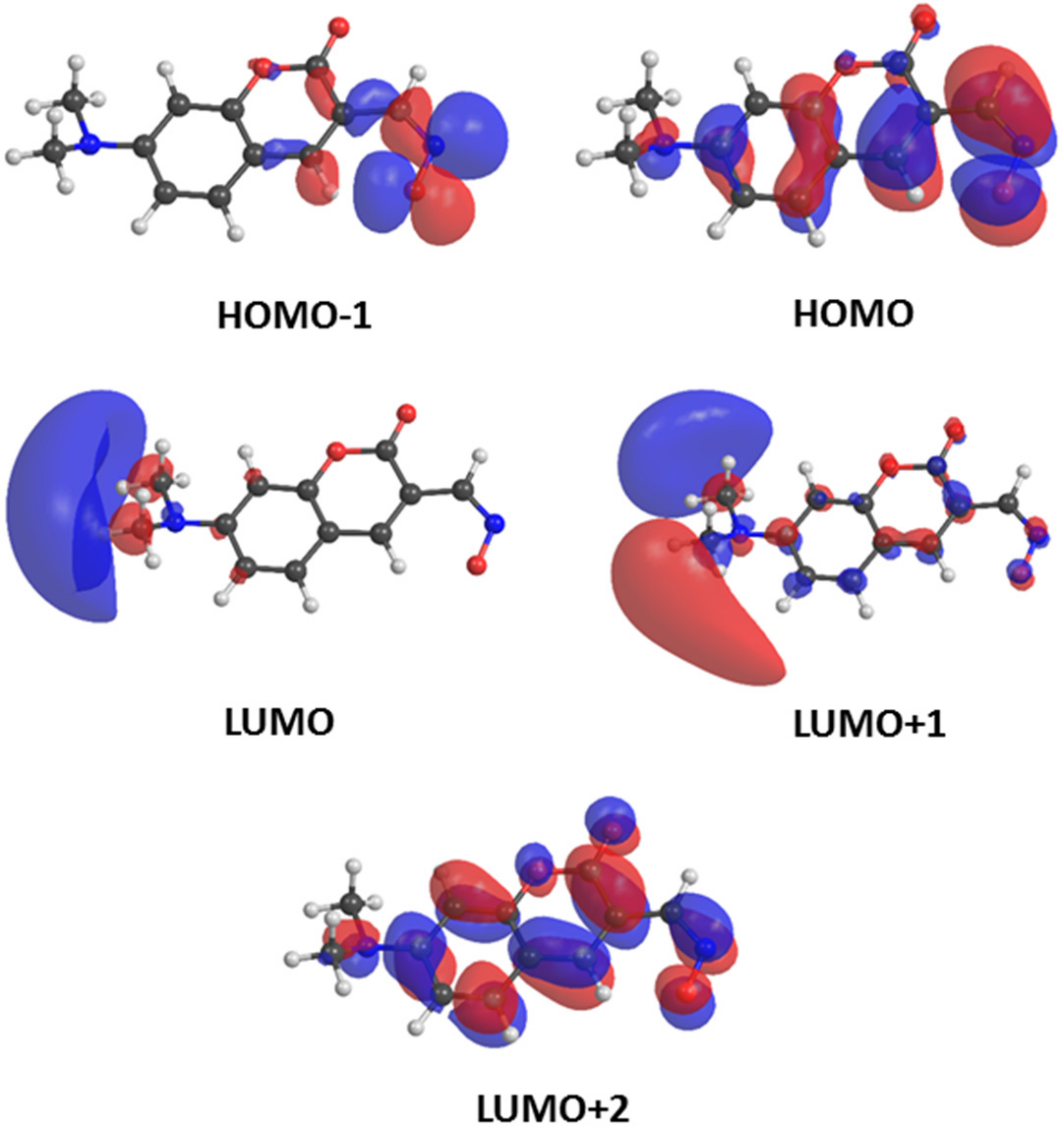
Molecular orbitals of most stable 7-dimethylaminocoumarin oximate **1** conformer (unusual shape of LUMO and LUMO+1 results mainly from combination of N(CH_3_)_2_ carbon 5s and 4p atomic orbitals; N(CH_3_)_2_ nitrogen 5s orbital also significantly contributes to LUMO molecular orbital).

Our quantum chemical calculations support the presence of dark S_1_ excited state [41] with almost zero oscillator strength related to S_0_ → S_1_ excitation process of oximate **1** (TD-DFT; M06-2X/311+G(2d,p); Figure 4 and Table 2). This excitation is represented dominantly by HOMO−1 to LUMO+1 and LUMO+2 transitions (Table 2; Figure 5). The HOMO−1 molecular orbital of oximate is a combination of almost 2p and 3p atomic orbitals of oxime nitrogen and oxygen (TD-DFT; M06-2X/311+G(2d,p)).

Nature bond orbital (NBO) analysis of oximate **1** shows single bond between these two atoms and presence of four lone electron pairs with significant p-orbital contributions on both atoms (ESI Table S3—yellow mark). One lone pair is localized on N16 and the other three on O17 atoms. Therefore, we assume that the dark S_1_ excited state of oximate **1** has significant *n*-π* character. This dark state can deactivate through internal conversion (ic) to the S_0_ ground state or by intersystem crossing (isc) to the triplet T_2_ excited state (Figure 4).

However, absence of any excited state absorption (ESA) signal in the transient absorption spectrum of oximate **1** using nanosecond flash photolysis system favours S_1_ excited state deactivation by internal conversion (ESI Figure S12). Contrary to aldoxime base, the lowest S_1_ excited state of initial oxime **1** has high oscillator strength and deactivates dominantly by a radiative (fluorescent) pathway (ESI Table S4; ESI Figure S13).

Calculated detection (3σ/S) limits for water using the studied two-component colorimetric and fluorescence “turn-on” 7-dialkylaminocoumarin oxime/F^−^ chemosensors **1**/F^−^ and **2**/F^−^ are amongst the lowest published detection limits for water in MeCN and DMF and chemosensor **1**/F^−^ in particular can compete in sensitivity with chemodosimeters or chemosensors based on interpolymer π-stacking aggregation (Table 3) [24]. Compared to other “competitive ligand-based” chemosensors for water content in aprotic polar solvents, determined 3σ/S in MeCN for **1**/F^−^ is even lower than that of the most sensitive Eu^3+^ luminiscent chemosensor published by Song et al. [42].

**Figure 6:**
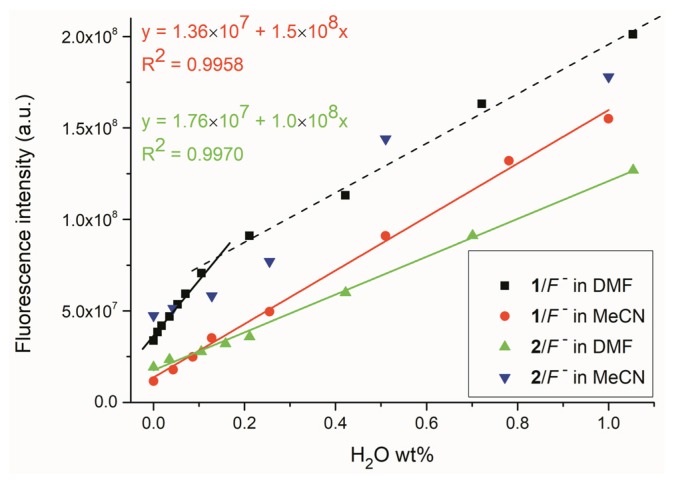
Fluorescence intensity behaviour of studied two-component coumarin oxime sensors **1**/F^−^ and **2**/F^−^ in MeCN and DMF during titration with water (λ_EX_ = λ_A(oxime)_; *T* = 298.15 K).

The valid water detection range for chemosensors **1**/F^−^ and **2**/F^−^ lies in the interval of 0–4 wt % and shows a good linearity in the range of 0–1 wt % for **1**/F^−^ in MeCN and **2**/F^−^ in DMF (Figure 6). Significant deviation from linearity was observed for chemosensor **2**/F^−^ in MeCN due to its slow thermal isomerization in this solvent. Chemosensor **1**/F^−^ exhibits rather double-linear behaviour in DMF and achieves high sensitivity particularly in 0–0.1 wt % region (ESI Figure S14).

Contrary to the significant fluorescence quenching of coumarin phenylsemicarbazones by basic CH_3_COO^−^ anions, neither other basic anions (such as CH_3_COO^−^, Br^−^, Cl^−^) nor excess Zn^2+^ cation influence the shape/spectral intensity of the studied coumarin oximes (ESI Figure S15).

However, addition of alcohols or acids to the coumarin oxime/F^−^ two-component system gives a similar response as the water addition (ESI Figures S16 and S17). Therefore the studied oximate chemosensors cannot be used for low-level water content determination in polar protic solvents or under acidic conditions. Although the sensing mechanism is based on a fast reversible acid-base equilibrium, sensor recovery will require oxime immobilization or its incorporation into a polymer matrix [43].

**Figure 7:**
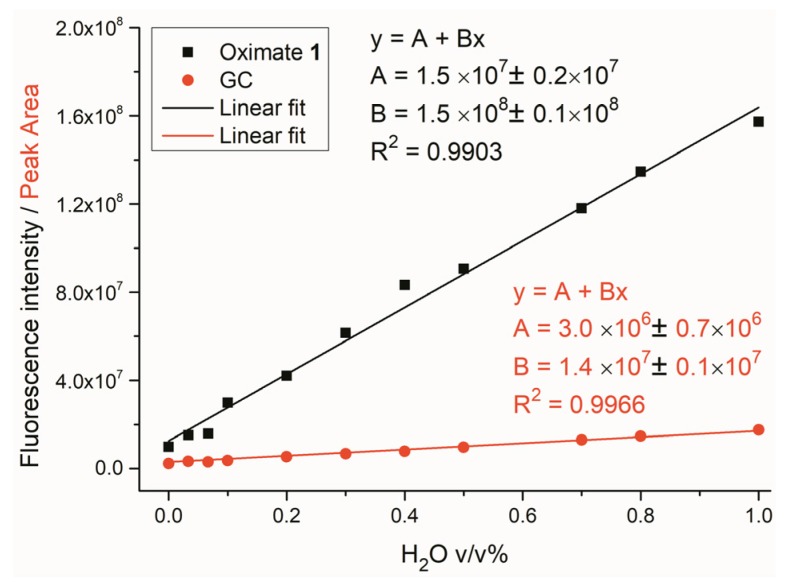
Calibration curves for low-level water content determination in acetonitrile using investigated oxime **1** based fluorescent “turn-on” chemosensor and gas chromatography with mass spectrometry (GC-MS) detection (capillary column coated with a 0.2 μm film thickness of ionic liquid SPB-IL100 as stationary phase was used; *v/v* (%)—volume of water/volume of solution in %).

To consider the benefits of our oxime based fluorescent “turn-on” method, we compared the calibration curve for water detection in acetonitrile with those obtained by ionic liquid-based gas chromatography with mass spectrometry (GC-MS) detection (Figure 7 and ESI Figure S18; the same H_2_O/MeCN calibration solutions were used). As shown in Figure 7, oxime chemosensors **1**/F^−^ gives steeper response to low-level water content compared to GC-MS method, however, GC-MS offers more universal application, small sample volume analysis and low-level water detection also in polar protic solvents.

## 3. Materials and Methods

### 3.1. General Information

All chemicals were purchased from Sigma-Aldrich (St. Louis, MO, USA). FTIR spectra were recorded on a Nicolet iS10 FT-IR instrument using the ATR technique (Thermo Scientific, Waltham, MA, USA) Elemental analyses were obtained on an Elementar vario MICRO cube (Elementar Analysensysteme GmbH, Langenselbold, Germany). Melting points were recorded on an IA-9200 Kofler apparatus (Cole-Parmer, Stone, United Kingdom). NMR spectra were recorded in 5 mm NMR tubes on a Varian VNMRS 600 MHz spectrometer (600 MHz for ^1^H and 150 MHz for ^13^C, Varian, Inc. Palo Alto, CA, USA) in DMSO-d_6_, with tetramethylsilane (TMS) as an internal standard.

Poly (propylene carbonate) thin polymer films of pure 7-dimethylaminocoumarin oxime **1** and two-component 7-dimethylaminocoumarin oxime **1**/F^−^ system were prepared by casting 1 mL chloroform solution of polymer (11 g/100 mL) containing the appropriate amount of oxime **1** (or oxime **1** + tetrabutylammonium fluoride) onto a large Teflon plate. The solvent was evaporated slowly. Remaining solvent was not additionally removed from the polymer. Poly (propylene carbonate) was purchased from Sigma-Aldrich, Mn ~ 50,000 by GPC). Final concentration of oxime **1** in chloroform solution was ~ 5 × 10^−4^ mol dm^−3^.

Gas chromatography with mass spectrometry (GC-MS) was used as a comparative method for low-level water content determination in acetonitrile. GC-MS measurements were performed on a 6890N gas chromatograph with a 5973 Network mass-selective detector (Agilent Technologies, Palo Alto, CA, USA). The injection port was maintained at 250 °C, and 1 μL of sample was injected with split ratio 10:1. The samples were separated using a 30 m capillary column with 0.25 mm inside diameter, coated with a 0.2 μm film thickness of ionic liquid SPB-IL100 (1,9-di(3-vinylimidazolium)nonane bis(trifluoromethylsulfonyl)imide) as stationary phase (Supelco, Bellefonte, PA, USA). The column temperature was 40 °C initially for 3 min, then increased to 70 °C at a ramp rate of 5 °C min^−1^. Helium carrier gas with a constant flow of 1.9 mL min^−1^ was used. The transfer line temperature was set at 300 °C. The mass spectrometer detector conditions were 2.5 min solvent delay, electron energy of 70 eV, ion source temperature of 230 °C and SIM acquisition mode with 17 and 18 *m*/*z* for water. The data handling was carried out using the MSD ChemStation software E.02.02 (Agilent Technologies).

### 3.2. Synthesis

#### 3.2.1. Synthesis of Coumarin Oximes **1** and **2**

*Method A*: 7-dimethylamino-2-oxo-2*H*-chromene-3-carbaldehyde or 10-oxo-2,3,5,6-tetrahydro-1*H*, 4*H*,10*H*-11-oxa-3a-aza-benzo[*de*]anthracene-9-carbaldehyde (1 mmol) and hydroxylamine dihydro-chloride (0.53 g, 5 mmol) were refluxed in ethanol (20 mL) for 24 h. The reaction mixture was then cooled to 0 °C and the solid residue was filtered, washed with cold ethanol (3 × 10 mL), recrystalized from boiling methanol (2 × 50 mL) and air dried. The solids were obtained in 96–98% yields.

*Method B*: By analogy with a literature procedure [44], hydroxylamine hydrochloride (0.07 g, 64.0 mmol) and Et_3_N (0.26 mL, 2 mmol) or NaOH (0.08 g, 2 mmol) were added to a solution of 7-dimethylamino-2-oxo-2*H*-chromene-3-carbaldehyde (0.22 g, 1 mmol) or 10-oxo-2,3,5,6-tetrahydro-1*H*, 4*H*,10*H*-11-oxa-3a-aza-benzo[*de*]anthracene-9-carbaldehyde (0.27 g, 1 mmol) in EtOH (30 mL). After stirring for 1 h, the reaction mixture was concentrated under reduced pressure to give a residue to which H_2_O (25 mL) was added, followed by extraction with dichloromethane (4 × 50 mL). The combined organic layers were washed with brine, dried over anhydrous NaSO_4_, filtered, and evaporated to give the crude product, which was recrystallized in *n*-pentane-EtOAc (1:1) to give the target oximes.

After treatment of the reaction mixture, the presence of starting aldehydes was observed in the corresponding NMR spectra. We assume that the oximes undergo hydrolysis to the corresponding aldehydes and hydroxylamine. The final product was also chromatographed on silicagel with dichloromethane as eluent, but the aldehydes were still present. Even six-fold crystallization from dry, polar aprotic acetonitrile, followed by preparative thin layer chromatography was not effective. We also tried to purify oximes by chromatography over a neutral alumina layer, but the result of this separation was even worse than in the case of chromatography on silicagel. We anticipate the presence of starting aldehydes (with relatively strong intermolecular bonding) derived from oxime hydrolysis. Final purity of oxime **1** and **2** based on ^1^H-NMR was 98% and 93%, respectively.

#### 3.2.2. Product Characterization

*7-Dimethylamino-2-oxo-2H-chromene-3-carbaldehyde oxime* (**1**). Yield 98% (purity 98%); yellow solid, m.p. 244–246 °C, Anal. Calcd. for C_12_H_12_N_2_O_3_ (232.24) C, 62.06; H, 5.21; N, 12.06. Found: C, 62.04; H, 5.20; N, 12.06. ^1^H-NMR (DMSO-*d*_6_) δ: 11.32 (s, 1H, OH, exchanged with D_2_O), 8.18 (s, 1H), 7.99 (s, 1H), 7.57 (d, 1H, *J* = 8.9 Hz), 6.76 (dd, 1H, *J* = 8.9, 2.3 Hz), 6.58 (d, 1H, *J* = 2.3 Hz), 3.05 (s, 3H, CH_3_) ppm. ^13^C-NMR (DMSO-*d*_6_) δ: 160.10, 155.79, 153.24, 142.9, 138.42, 129.97, 112.04, 109.79, 108.16, 96.99, 40.07 ppm. IR (ATR, cm^−1^): 3196 (O-H), 1701 (C=O), 1619 and 1520 (C=C and C=N). TLC (dichloromethane): *R*_f_ = 0.1.

*10-Oxo-2,3,5,6-tetrahydro-1H,4H,10H-11-oxa-3a-aza-benzo[de]anthracene-9-carbaldehyde oxime* (**2**). Yield 96% (purity 93%); orange solid, m.p. 257–259 °C, Anal. Calcd. for C_16_H_16_N_2_O_3_ (284.31) C, 67.59; H, 5.67; N, 9.85. Found: C, 67.56; H, 5.65; N, 9.85. ^1^H-NMR (DMSO-*d*_6_) δ: 11.22 (s, 1H, OH, exchanged with D_2_O), 8.03 (s, 1H), 7.98 (s, 1H), 7.13 (s, 1H), 1.85–1.91 (m, 4H, 2× CH_2_), 2.69–2.74 (m, 4H, 2× CH_2_-N), 3.25–3.30 (m, 4H, 2× CH_2_-A_R_) ppm. ^13^C-NMR (DMSO-d_6_) δ: 160.75, 151.58, 146.85, 143.41, 138.89, 126.56, 119.17, 110.79, 108.03, 105.67, 49.83, 49.32, 27.26, 21.20, 20.29, 20.14 ppm. IR (ATR, cm^−1^): 3327 (O-H), 2934 (N-CH_2_), 2840 (N-CH_2_), 1663 (C=O), 1513 and 1435 (C=C and C=N). TLC (dichloromethane): *R*_f_ = 0.2.

The corresponding ^1^H-, ^13^C- and 2D NMR spectra are shown in ESI Figures S19 and S20.

### 3.3. Spectroscopic Measurements

Electronic absorption spectra were obtained on a HP 8452A diode array spectrophotometer (Hewlett Packard, Palo Alto, CA, USA). The solvents used (MeCN, DMSO, DMF) were HPLC (MeCN; LiChrosolv ^®^, Merck, Darmstadt, Germany) or UV-spectroscopy grade (DMSO and DMF; Uvasol ^®^, Merck, Darmstadt, Germany) and were used without further purification. Solution fluorescence was measured in a 1 cm cuvette with a FSP 920 (Edinburgh Instruments, Edinburgh, United Kingdom) spectrofluorimeter in a right-angle or front-face arrangement (to exclude solution self-absorption). The fluorescent quantum yield (Φ_F_) of studied compounds in solution was determined by Equations (1) and (2) using integrating sphere (Edinburgh Instruments):

(1) $$\Phi_{F}^{X}=\frac{L_{\mathrm{Sam}}}{E_{\mathrm{Ref}}-E_{\mathrm{Sam}}}\left(\%\right)$$

corrected to re-absorption by:

(2) $$\Phi_{F}=\frac{\Phi_{F}^{X}}{1-a+a\Phi_{F}^{X}/100}\;\left(\%\right)$$

where *L*_Sam_ is the area under the detected spectrum in the part of the spectrum where sample emission occurs, *E*_Ref_ is the area under the reflection part of the detected spectrum using pure solvent as reference material (diffuse reflectance), *E*_Sam_ is the area under the reflection part of the detected spectrum after absorption by sample and a is reabsorbed area. The time-resolved fluorescence measurements were performed on a FSP 920 spectrofluorimeter (Edinburgh Instruments) with a time-correlated single-photon counting (TCSPC) module and a red sensitive high speed photomultiplier in peltier housing, featuring Hamamatsu H5773-04 detector (R928P detector; Edinburgh Photonics, Edinburgh, United Kingdom). Excitation source was 402.8 nm picosecond pulsed diode laser (Model EPL-405; Pulse Width: 60.5 ps; Edinburgh Photonics). Reconvolution fit analysis software (F900, Edinburgh Instruments) was used for lifetime data analysis. Transient absorption spectra of oximate **1** were measured on a Flash photolysis LP980-Spectrometer (Edinburgh Instruments).

### 3.4. Titration Experiments

#### 3.4.1. Materials

In the titration experiments all four anions (F^−^, CH_3_COO^−^, Br^−^ and Cl^−^) were added in the form of the corresponding tetrabutylammonium (TBA^+^) salts purchased from Sigma-Aldrich and used without further purification. Zn^2+^ cation was added in the form of ZnCl_2_ (Sigma-Aldrich). Distilled water was used in all H_2_O titration experiments.

#### 3.4.2. General Method

All titration experiments were carried out in MeCN (or in DMF) at 298.16 K. The coumarin oxime solutions were titrated with distilled water to obtain 5 × 10^−5^ mol·dm^−3^ overall coumarin oxime concentrations in the resultant solution. The titration process was monitored by UV-Vis (HP 8452A, Hewlett Packard, Palo Alto, CA, USA) and fluorescence (FSP 920, Edinburgh Instruments, Edinburgh, United Kingdom) spectroscopy (using a 1 cm cuvette).

#### 3.4.3. Detection and Quantification Limits

Detection limit (3σ/S) and quantification limit (10σ/S) for water in MeCN were determined by the following equations:

(3) $$3\sigma /S=3\frac{\sqrt{\frac{\Sigma {\left(F-\bar{F}\right)}^{2}}{n-1}}}{S};$$

(4) $$10\sigma /S=10\frac{\sqrt{\frac{\Sigma {\left(F-\bar{F}\right)}^{2}}{n-1}}}{S},$$

where: *σ* is the standard deviation, *F* is the area under the fluorescence emission curve of coumarin oxime and *S* is the slope of the *F* = *f*(wt %) or *F* = *f*(*v/v* %) plot in the initial linear portion of the curve; in all cases *n* = 5.

### 3.5. Quantum-Chemical Calculations

The Gibbs free energy of the 7-dimethylaminocoumarin oxime/oximate conformers, natural bond orbital (NBO) analysis [45,46,47], excitation energies and oscillator strength of most stable conformers were investigated using quantum-chemical calculations at the M06-2X/6-311+G(2d,p) level of theory. Geometries were optimized at the M06-2X/6-31+G(d,p) level of theory. Stationary points were characterized as minima by computation of harmonic vibrational frequencies. All calculations were performed by Gaussian 09 program package [48].

## 4. Conclusions

This paper investigated the sensing mechanism, fluorescent response pathways and sensitivity of two new two-component fluorescent “turn-on” chemosensors based on the 7-dialkyl-aminocoumarin oxime acid-base equilibrium. Addition of strongly basic anions to the coumarin oxime solutions leads to the deprotonation of the most acidic =N-O-H hydrogen and almost complete fluorescence quenching based on the dark S_1_ excited state population in conjugate oximates. However, traces of water already result in intense fluorescence recovery of the initial oxime due to back aldoxime base protonation. Polar protic solvents compete with water as base in an acid-base equilibrium and the studied chemosensors therefore cannot be used for low-level water content determination in these solvents. To the best of our knowledge, the determined detection limits for water by the studied chemosensors are amongst the lowest detection limits ever published in the literature and can compete in sensitivity with chemodosimeters or chemosensors based on interpolymer π-stacking aggregation. Their deficiency is, however, the necessity of F^−^ base presence.

## Figures and Tables

**Table 1:** Basic spectral characteristics of studied coumarin oximes **1** and **2** in MeCN, DMF and DMSO.

Compd.	λ_A_ (nm)	Log ε_A_	λ_F_ (nm)	Φ_F_	τ (ns)	χ^2^
MeCN						
**1**	413	4.27	487	0.80	τ_1_ = 1.6 (10%)	1.111
					τ_2_ = 3.5 (90%)	
**2**	438	4.28	504	0.87	τ_1_ = 0.5 (2%)	1.050
					τ_2_ = 3.6 (98%)	
DMF						
**1**	414	4.52	491	0.87	τ_1_ = 0.3 (3%)	1.171
					τ_2_ = 3.3 (97%)	
**2**	442	4.55	507	0.95	τ_1_ = 0.5 (2%)	1.196
					τ_2_ = 3.6 (98%)	
DMSO						
**1**	419	4.54	497	0.88	τ_1_ = 1.5 (8%)	1.007
					τ_2_ = 3.3 (92%)	
**2**	442	4.51	512	1.00	τ = 3.6 (100%)	1.130

λ_A_—Long-wavelength absorption maximum; Log ε_A_—Log of the molar extinction coefficient in λ_A_; λ_F_—Fluorescence maximum; Φ_F_—Fluorescent quantum yield; τ—Fluorescence/excited state lifetime (relative fluorescence intensity of the corresponding component); χ^2^—Quality of fluorescence lifetime fitting.

**Table 2:** Excitation energies, oscillator strengths (*f*) and orbital contributions to corresponding electronic transitions from ground state (S_0_) of oximate **1** (geometry was optimized at the M06-2X/6-31+G(d,p) level of theory and energies were calculated at the M06-2X/6-311+G(2d,p) level of theory).

7-Dimethylaminocoumarin Oximate 1				
Excited State	Orbital Contributions	Energy		Oscillator Strength
		[eV]	[nm]	
T_1_	HOMO → LUMO (2%)	1.31	946	0.0000
	HOMO → LUMO+1 (10%)			
	HOMO → LUMO+2 (77%)			
T_2_	HOMO−1 → LUMO+1 (7%)	1.36	914	0.0000
	HOMO−1 → LUMO+2 (54%)			
S_1_	HOMO−1 → LUMO+1 (8%)	1.80	686	0.0003
	HOMO−1 → LUMO+2 (61%)			
S_2_	HOMO → LUMO (12%)	3.06	406	0.5360
	HOMO → LUMO+1 (10%)			
	HOMO → LUMO+2 (73%)			

* Transitions to higher molecular orbitals than LUMO+2 are not shown.

**Table 3:** Detection limit (LOD) and quantification limit (LOQ) for water in MeCN and DMF using studied two-component coumarin oxime/F^−^ chemosensors (determined by fluorescence spectroscopy; *T* = 298.16 K).

	LOD (3σ/S)		LOQ (10σ/S)	
	wt %	*v/v* %	wt %	*v/v* %
MeCN				
**1**/F^−^	0.0014	0.0012	0.0048	0.0039
**2**/F^−^	0.0175 *	0.0137 *	0.0583 *	0.0457 *
DMF				
**1**/F^−^	0.0059	0.0046	0.0196	0.0153
**2**/F^−^	0.0046	0.0044	0.0154	0.0146

wt (%)—weight of water/weight of solution in %; *v/v* (%)—volume of water/volume of solution in %; *—standard deviation is probably influenced by slow thermal isomerization.

## Supplementary Materials

The following are available online at www.mdpi.com/1420-3049/22/8/1340/s1, Figure S1: Evolution of the fluorescence spectrum of 7-dimethylaminocoumarin oxime **1** in MeCN after F^−^ anion and subsequent water addition; Figure S2: Evolution of the fluorescence spectrum of 7-dimethylaminocoumarin oxime **1** in DMF after F^−^ anion and subsequent water addition; Figure S3: Evolution of the fluorescence spectrum of 7-coumarin oxime **2** in MeCN after F^−^ anion and subsequent water addition; Figure S4: Evolution of the fluorescence spectrum of coumarin oxime **2** in DMF after F^−^ anion and subsequent water addition; Figure S5: Evolution of the emission spectrum of 7-coumarin oxime **1** in MeCN during **1** solution titration with TBA^+^F; Figure S6: Evolution of the emission spectrum of coumarin oxime **2** in MeCN during **2** solution titration with TBA^+^F^−^; Figure S7: Evolution of fluorescence decay of 7-coumarin oxime **1** in MeCN during **1** solution titration with TBA^+^F^−^; Figure S8: ^1^H NMR spectrum of 7-dimethylaminocoumarin oxime **1** in DMSO-*d*_6_ before and after F^−^ anion (TBA^+^F^−^) and subsequent water addition; Figure S9: ^1^H-NMR spectrum of coumarin oxime **2** in DMSO-d_6_ before and after F^−^ anion (TBA^+^F^−^) and subsequent water addition; Figure S10: Geometries of oxime **1**/oximate **1** stable conformers optimized at the M06-2X/6-31+G(d,p) level of theory; Figure S11: Poly(propylene carbonate) thin polymer films of pure 7-dimethylaminocoumarin oxime **1** and two-component 7-dimethylaminocoumarin oxime **1**/F^−^ system on teflon plate; Figure S12: Transient absorption spectrum of oximate **1** in MeCN, Table S4: Excitation energies, oscillator strengths (*f*) and orbital contributions to corresponding electronic transitions from ground state (S_0_) of studied coumarin oxime **1**; Figure S13: Frontier molecular orbitals of 7-dimethylaminocoumarin oxime **1**; Figure S14: Fluorescence intensity behaviour of studied two-component coumarin oxime sensor **1**/F^−^ in DMF during titration with water; Figure S15: Fluorescence spectrum of 7-dimethylaminocoumarin oxime **1**/F^−^ system in MeCN after 4 wt % water addition in the presence of various ion excess; Figure S16: Evolution of the emission spectrum of 7-dimethylaminocoumarin oxime **1**/F^−^ system in MeCN during **1**/F^−^ solution titration with methanol; Figure S17: Evolution of the emission spectrum of 7-dimethylaminocoumarin oxime **1**/F^−^ system in MeCN during **1**/F^−^ solution titration with acetic acid; Figure S18: GC-MS chromatogram of water analysis in MeCN in ionic liquid capillary column; Figure S19: Coumarin oxime **1**: ^1^H-NMR, ^13^C-NMR, HSQC and HMBC; Figure S20: Coumarin oxime **2**: ^1^H-NMR, ^13^C-NMR, HSQC and HMBC, Scheme S1: Molecular structure of compounds **4**–**6** and scheme of possible intramolecular photoinduced electron transfer (PET), Table S1: Relative Gibbs free energy (∆G) of oxime **1**/oximate **1** stable conformers calculated at the M06-2X/6-311+G(2d,p) level in vacuum; Table S2: Calculated HOMO and LUMO orbital energy of compounds **3**–**6** at the M06-2X/6-31+G(d,p) level in vacuum; Table S3: Natural bond orbital (NBO) analysis of oximate **1**.
