# Supplementary material for: 7-Dialkylaminocoumarin Oximates: Small Molecule Fluorescent “Turn-On” Chemosensors for Low-Level Water Content in Aprotic Organic Solvents
Source: Molecules. 2017 Aug 12;22(8):1340. doi: 10.3390/molecules22081340 (PMC6152144; doi:10.3390/molecules22081340)

Supplementary Materials: Electronic Supplementary Information (ESI)

7-Dialkylaminocoumarin Oximates: Small Molecule Fluorescent "Turn-on" Chemosensors for Low-Level Water Content in Aprotic Organic Solvents

Marek Cigáň [1,*], Miroslav Horváth [1], Juraj Filo [1], Klaudia Jakusová [1], Jana Donovalová [1], Vladimír Garaj [2] and Anton Gáplovský [1]

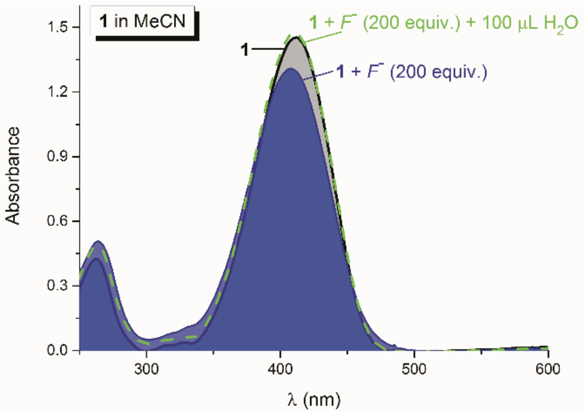

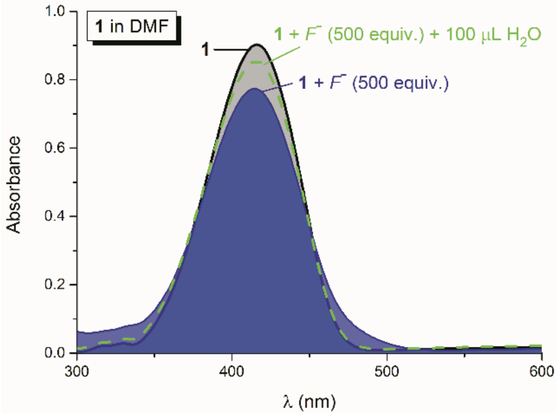

Fig. S1. (left) Evolution of the absorption spectrum of 7-dimethylaminocoumarin oxime **1** in MeCN after F^- anion and subsequent water addition (5×10^{-5} mol L^{-1} **1** + 1×10^{-2} mol L^{-1} TBA^+F^-; $T = 298.15$ K).

Fig. S2. (right) Evolution of the absorption spectrum of 7-dimethylaminocoumarin oxime **1** in DMF after F^- anion and subsequent water addition (2×10^{-5} mol L^{-1} **1** + 1×10^{-2} mol L^{-1} TBA^+F^-; $T = 298.15$ K).

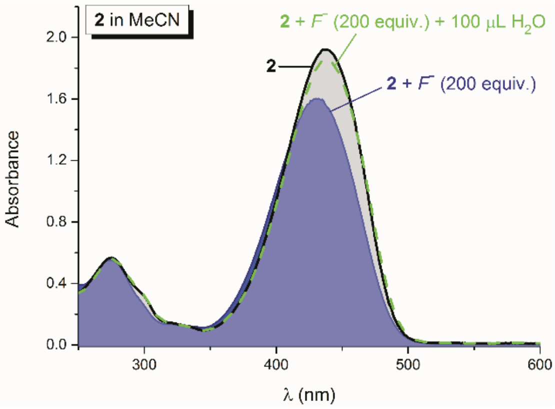

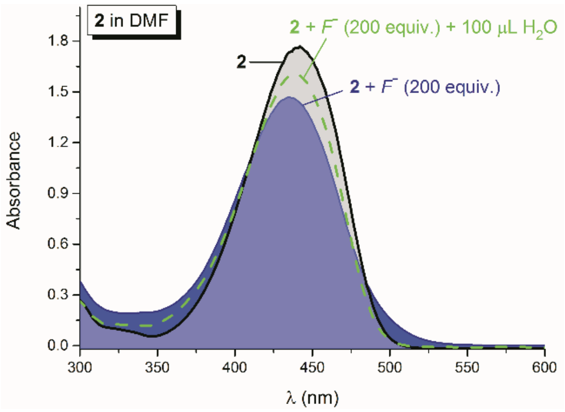

Fig. S3. (left) Evolution of the absorption spectrum of 7-coumarin oxime **2** in MeCN after F^- anion and subsequent water addition (5×10^{-5} mol L^{-1} **1** + 1×10^{-2} mol L^{-1} TBA^+F^-; $T = 298.15$ K).

Fig. S4. (right) Evolution of the absorption spectrum of coumarin oxime **2** in DMF after F^- anion and subsequent water addition (5×10^{-5} mol L^{-1} **1** + 1×10^{-2} mol L^{-1} TBA^+F^-; $T = 298.15$ K).

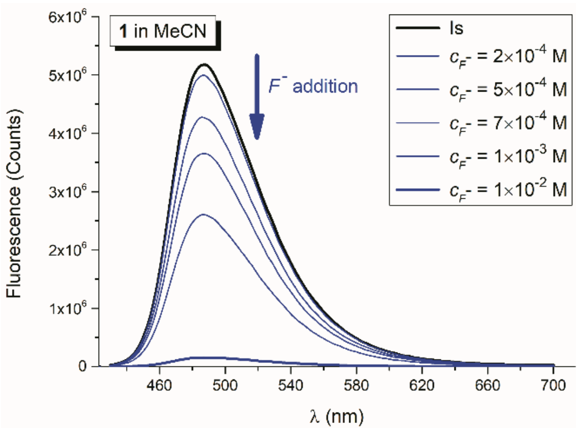

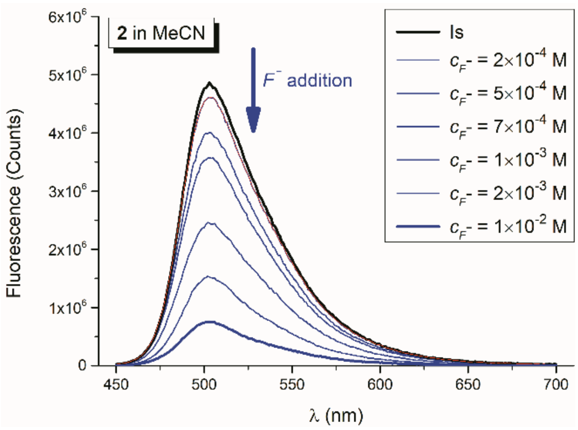

Fig. S5. (left) Evolution of the emission spectrum of 7-coumarin oxime **1** in MeCN during **1** solution titration with *TBA*$^+$*F* (5×10^{-5} mol L^{-1} **1**; Is – initial state; T = 298.15 K; M = mol dm^{-3}).

Fig. S6. (right) Evolution of the emission spectrum of coumarin oxime **2** in MeCN during **2** solution titration with *TBA*$^+$*F*$^-$ (5×10^{-5} mol L^{-1} **2**; Is – initial state; T = 298.15 K; M = mol dm^{-3}).

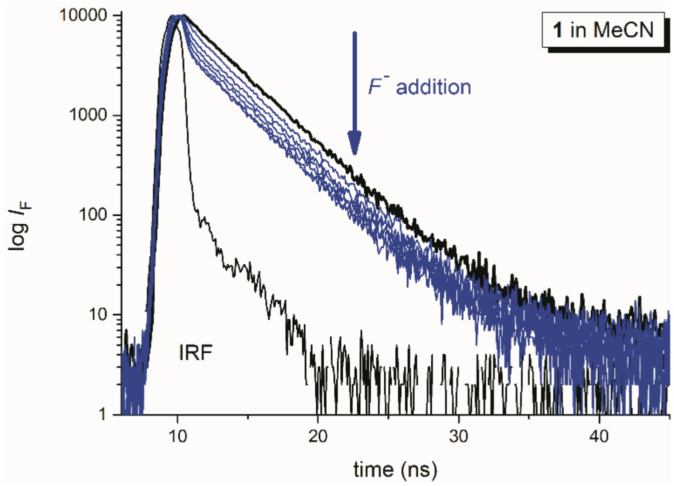

Fig. S7. (right) Evolution of fluorescence decay of 7-coumarin oxime **1** in MeCN during **1** solution titration with *TBA*$^+$*F*$^-$ (5×10^{-5} mol L^{-1} **1**; T = 298.15 K).

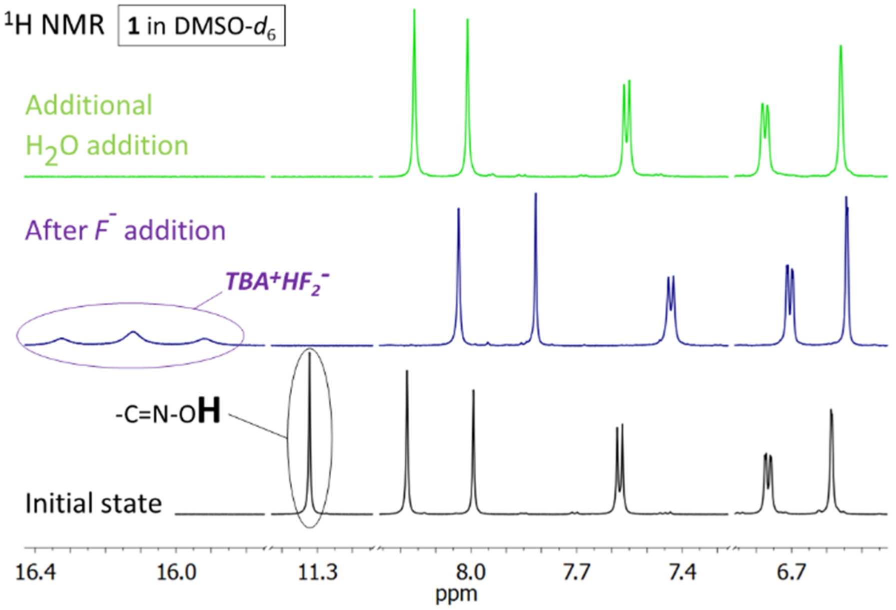

Fig. S8. ^{1}H NMR spectrum of 7-dimethylaminocoumarin oxime **1** in DMSO-d_6 before and after F^- anion (TBA^+F^-) and subsequent water addition ($c_1 = 5\times10^{-4}$ mol L^{-1}; $c_{F^-} = 1\times10^{-1}$ mol L^{-1}; 4% (v/v) of water; T = 298.15 K).

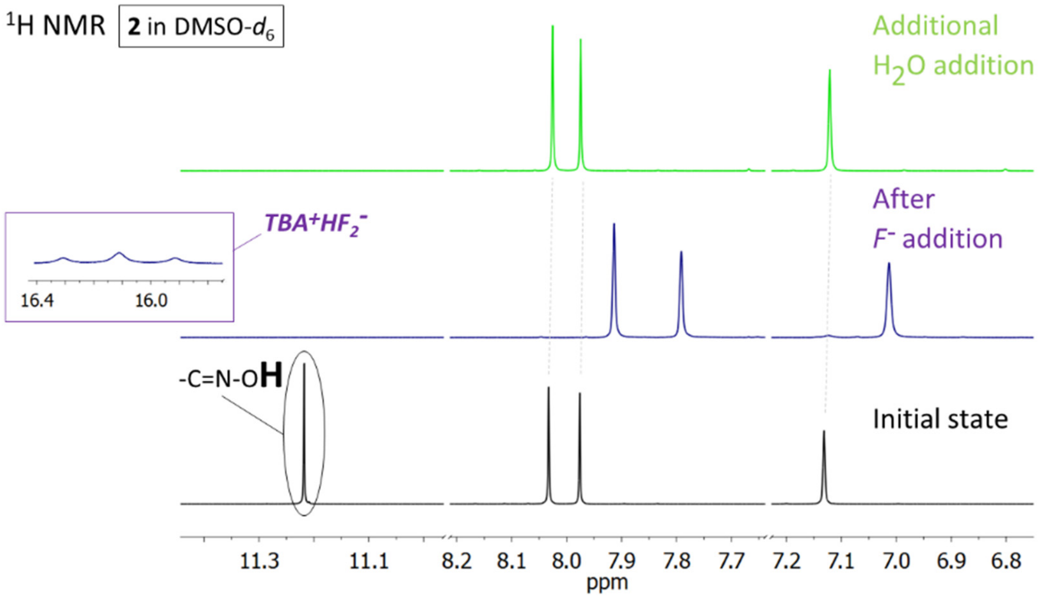

Fig. S9. ^{1}H NMR spectrum of coumarin oxime **2** in DMSO-d_6 before and after F^- anion (TBA^+F^-) and subsequent water addition ($c_2 = 5\times10^{-4}$ mol L^{-1}; $c_{F^-} = 1\times10^{-1}$ mol L^{-1}; 4% (v/v) of water; T = 298.15 K).

Table S1. Relative Gibbs free energy (ΔG) of oxime **1**/oximate **1** stable conformers calculated at the M06-2X/6-311+G(2d,p) level of theory in vacuum on geometries optimized at M06-2X/6-31+G(d,p) level of theory (calculated in Gaussian 09).

Conformer	ΔG (kJ mol^{-1})
oxime I	0
oxime II	2
oxime III	16
oxime IV	23
oxime V	40
oximate VI	0
oximate VII	6
oximate VIII	33
oximate IX	59

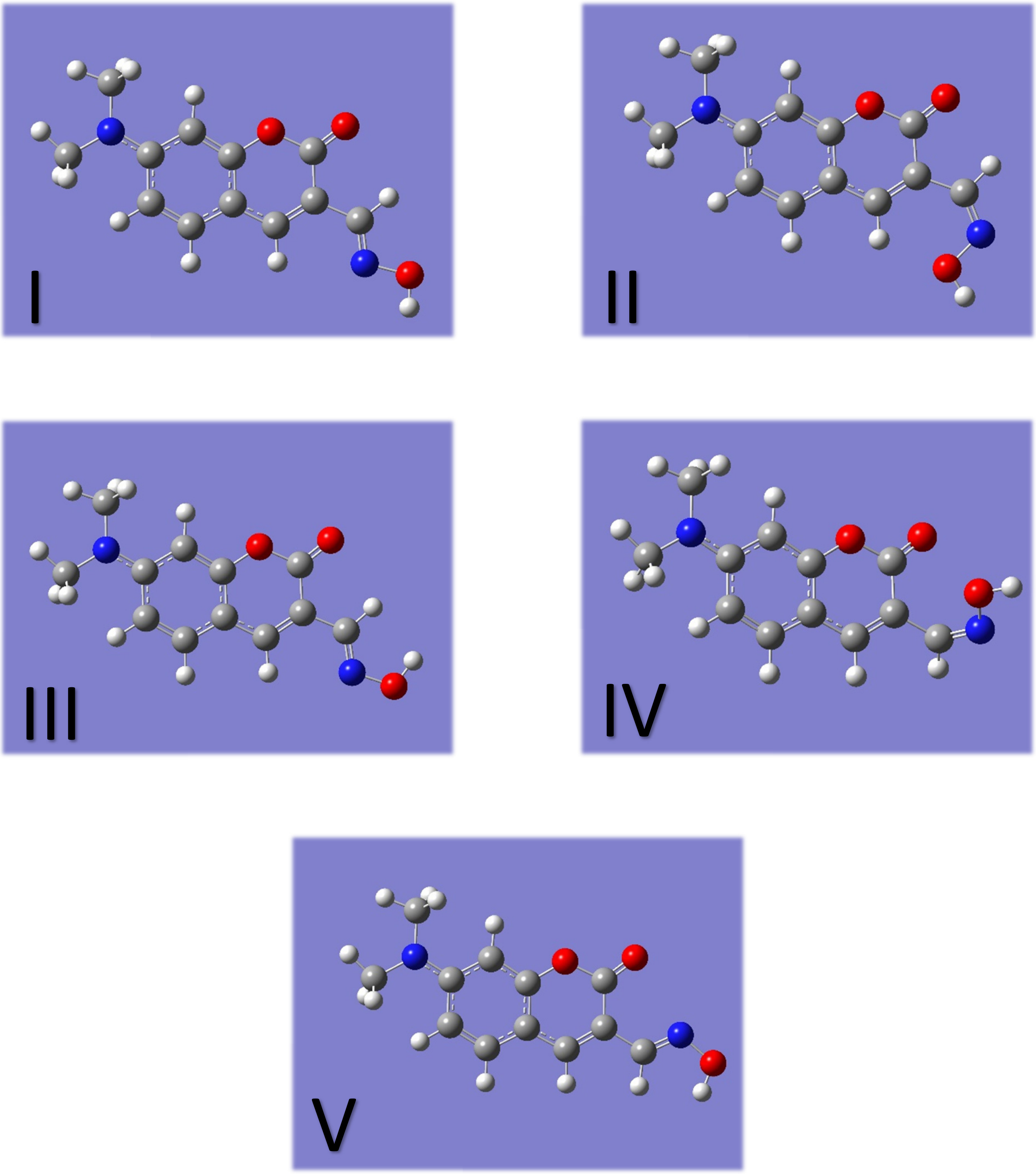

Fig. S10. Geometries of oxime **1** stable conformers optimized at the M06-2X/6-31+G(d,p) level of theory (calculated in Gaussian 09).

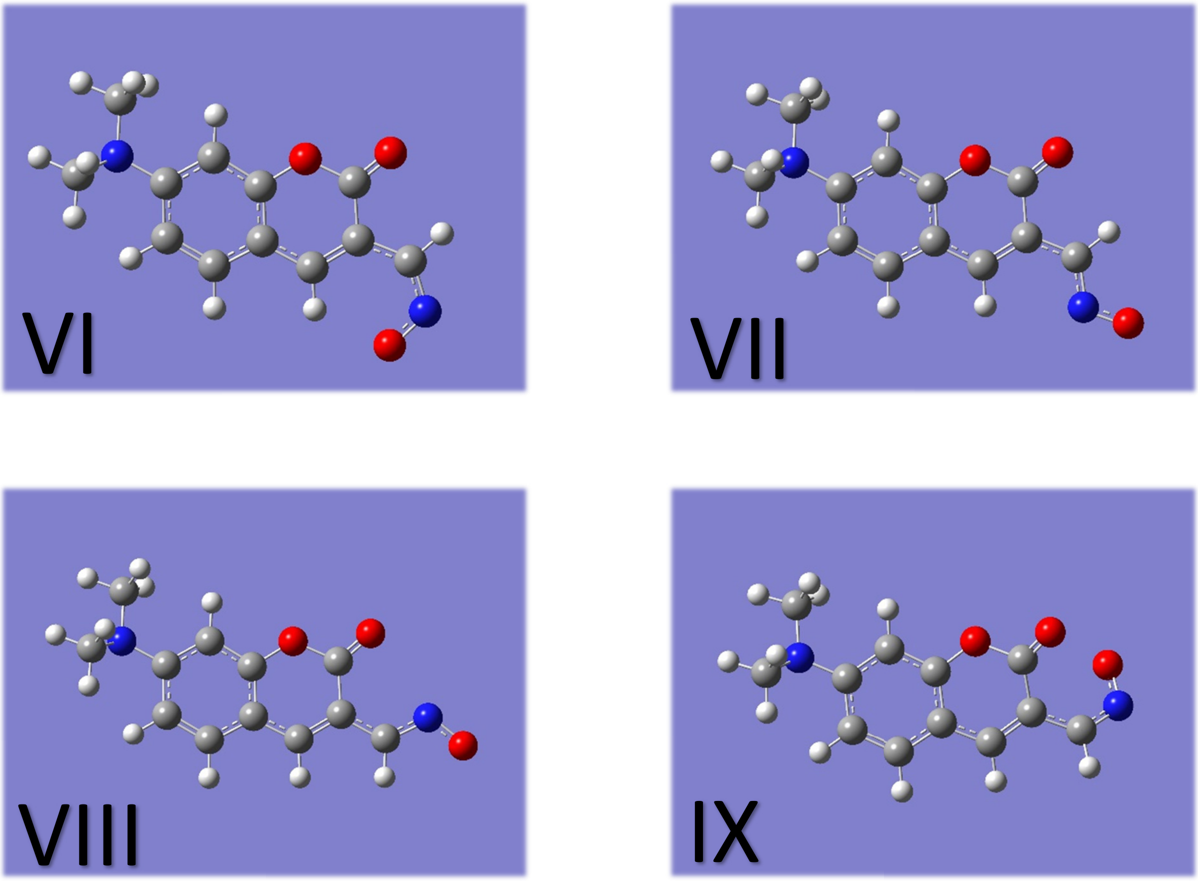

Fig. S10. Geometries of oximate **1** stable conformers optimized at the M06-2X/6-31+G(d,p) level of theory (calculated in Gaussian 09).

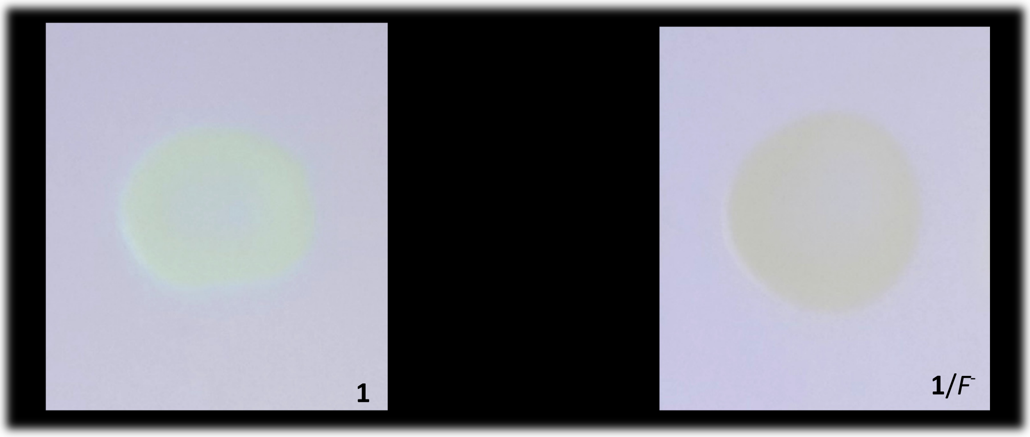

Φ_F = 0.80 Φ_F = 0.20

Fig. S11. Poly (propylene carbonate) thin polymer films of pure 7-dimethylaminocoumarin oxime **1** and two-component 7-dimethylaminocoumarin oxime **1**/*F*⁻ system on teflon plate (*TBA⁺F⁻* addition to chloroform solution of polycarbonate and oxime **1**; λ_{EX} = λ_A; Φ_F were determined using integrating sphere; poly (propylene carbonate) was purchased from Sigma-Aldrich, St. Louis, MO, USA, Mn ~ 50,000 by GPC).

Table S2. Calculated HOMO and LUMO orbital energy of compounds **3-6** at the M06-2X/6-31+G(d,p) level in vacuum (calculated in Gaussian 09).

Compd	3	4	5	6
**LUMO** (kJ mol^{-1})	-77	-75	8	343
**HOMO** (kJ mol^{-1})	-664	-645	-903	-91

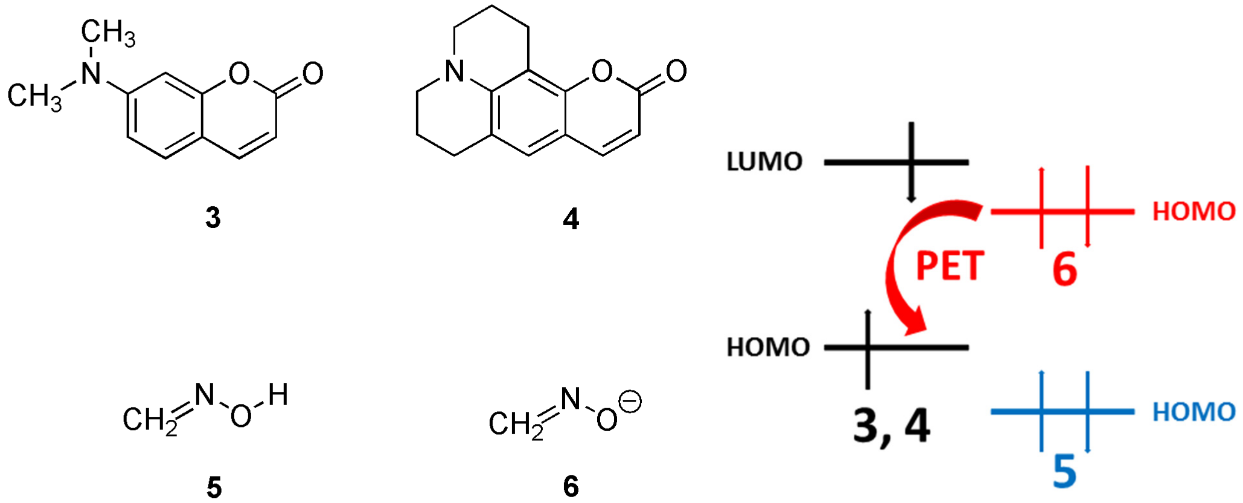

Scheme S1. Molecular structure of compounds **4-6** and scheme of possible intramolecular photoinduced electron transfer (PET).

Table S3. Natural bond orbital (NBO) analysis of oximate **1**.

NATURAL BOND ORBITAL ANALYSIS:

```
     (Occupancy)   Bond orbital/ Coefficients/ Hybrids
 -------------------------------------------------------------------------------

(1.99179) BD ( 1) C  14 - N  16
            ( 41.69%)   0.6457* C  14 s( 32.72%)p 2.05( 67.21%)d 0.00(  0.08%)
                                   0.0000  0.5708 -0.0372  0.0032 -0.0005
                                   0.4757 -0.0724  0.0058 -0.0018 -0.6622
                                   0.0402  0.0050  0.0018 -0.0197 -0.0006
                                   0.0005  0.0000 -0.0144 -0.0124  0.0003
                                  -0.0008  0.0017 -0.0003 -0.0057 -0.0033
                                  -0.0192 -0.0002
            ( 58.31%)   0.7636* N  16 s( 38.37%)p 1.60( 61.31%)d 0.01(  0.33%)
                                   0.0001  0.6182 -0.0368  0.0132 -0.0001
                                  -0.4918 -0.0131  0.0098  0.0018  0.6082
                                  -0.0215 -0.0163  0.0030  0.0156 -0.0018
                                  -0.0006  0.0003 -0.0488 -0.0018 -0.0005
                                  -0.0003  0.0005 -0.0001  0.0077 -0.0017
                                  -0.0290  0.0015


(1.80211) BD ( 2) C  14 - N  16
            ( 56.09%)   0.7489* C  14 s(  0.00%)p 1.00( 99.97%)d 0.00(  0.03%)
                                   0.0000 -0.0005  0.0000 -0.0001  0.0000
                                  -0.0411 -0.0021 -0.0007 -0.0002 -0.0597
                                  -0.0030 -0.0009 -0.0004  0.9958  0.0503
                                   0.0158  0.0063 -0.0001  0.0005  0.0082
                                  -0.0020 -0.0108 -0.0081 -0.0010 -0.0004
                                  -0.0005 -0.0010
            ( 43.91%)   0.6626* N  16 s(  0.00%)p 1.00( 99.72%)d 0.00(  0.28%)
                                   0.0000  0.0002 -0.0001  0.0001  0.0000
                                  -0.0424  0.0001 -0.0004  0.0002 -0.0600
                                   0.0000 -0.0007  0.0003  0.9958 -0.0012
                                   0.0116 -0.0046  0.0026 -0.0003 -0.0508
                                   0.0013  0.0117  0.0053  0.0028  0.0003
                                  -0.0025  0.0007


(1.99247) BD ( 1) N  16 - O  17
            ( 45.02%)   0.6710* N  16 s( 25.25%)p 2.95( 74.43%)d 0.01(  0.33%)
                                  -0.0001 -0.4957  0.0815  0.0120  0.0003
                                   0.3461  0.0040 -0.0098 -0.0043  0.7817
                                  -0.0965  0.0066 -0.0020  0.0620 -0.0057
                                   0.0000 -0.0003 -0.0426 -0.0076 -0.0046
                                  -0.0002 -0.0061 -0.0009  0.0119  0.0081
                                   0.0336  0.0010
```

(54.98%)   0.7415* O 17 s(26.03%)p 2.84(73.87%)d 0.00(0.10%)
-0.0001 -0.5057 0.0673 0.0022 0.0000
-0.3496 0.0290 -0.0047 -0.0003 -0.7808
0.0447 0.0059 -0.0030 -0.0625 0.0039
0.0001 -0.0002 -0.0200 -0.0032 -0.0014
-0.0003 -0.0035 -0.0007 0.0191 0.0039
0.0138 0.0032

(1.95729) LP (1) N 16   s(37.02%)p 1.70(62.82%)d 0.00(0.16%)
-0.0003 0.6063 0.0512 -0.0035 0.0002
0.7898 0.0452 0.0035 0.0047 0.0275
0.0159 0.0052 -0.0017 0.0352 0.0029
0.0005 0.0001 -0.0060 0.0039 -0.0034
0.0007 -0.0010 -0.0001 -0.0295 0.0069
0.0243 -0.0018

(1.98593) LP (1) O 17   s(74.14%)p 0.35(25.85%)d 0.00(0.00%)
-0.0002 0.8608 0.0221 0.0012 0.0000
-0.2601 0.0081 0.0062 -0.0002 -0.4350
0.0103 0.0007 -0.0030 -0.0376 0.0009
0.0003 -0.0002 -0.0042 -0.0010 -0.0004
-0.0001 -0.0008 -0.0002 0.0029 0.0013
0.0039 0.0010

(1.96420) LP (2) O 17   s(0.27%)p99.99(99.70%)d 0.11(0.03%)
-0.0001 0.0512 -0.0040 -0.0092 0.0000
0.8975 0.0094 -0.0120 -0.0017 -0.4369
-0.0135 -0.0056 -0.0009 0.0122 -0.0003
-0.0007 -0.0001 0.0133 -0.0019 0.0012
-0.0002 -0.0001 -0.0001 0.0111 -0.0014
0.0005 0.0010

(1.53924) LP (3) O 17   s(0.00%)p 1.00(99.91%)d 0.00(0.09%)
0.0000 -0.0001 0.0001 0.0000 0.0000
-0.0430 -0.0009 -0.0005 0.0001 -0.0605
-0.0013 -0.0008 0.0001 0.9964 0.0224
0.0151 -0.0020 -0.0017 0.0002 0.0084
-0.0005 0.0277 -0.0038 0.0013 -0.0002
0.0036 -0.0005

NATURAL POPULATIONS: Natural atomic orbital occupancies

NAO	Atom	No	lang	Type(AO)	Occupancy	Energy
267	C	14	S	Cor(1S)	1.99925	-10.24859
268	C	14	S	Val(2S)	0.90734	-0.00475
269	C	14	S	Ryd(3S)	0.00358	1.34642
270	C	14	S	Ryd(4S)	0.00020	3.96174
271	C	14	S	Ryd(5S)	0.00002	18.57657
272	C	14	px	Val(2p)	0.99817	0.11896
273	C	14	px	Ryd(4p)	0.00933	1.05646
274	C	14	px	Ryd(3p)	0.00096	0.74087
275	C	14	px	Ryd(5p)	0.00022	3.33408
276	C	14	py	Val(2p)	1.05382	0.09989
277	C	14	py	Ryd(4p)	0.01086	1.22127
278	C	14	py	Ryd(3p)	0.00073	0.58982
279	C	14	py	Ryd(5p)	0.00019	3.52857
280	C	14	pz	Val(2p)	1.17544	0.03370
281	C	14	pz	Ryd(4p)	0.00853	0.67088
282	C	14	pz	Ryd(3p)	0.00031	0.44905
283	C	14	pz	Ryd(5p)	0.00011	2.99884
284	C	14	dxy	Ryd(3d)	0.00062	2.27965
285	C	14	dxy	Ryd(4d)	0.00045	3.66097
286	C	14	dxz	Ryd(3d)	0.00022	1.87841
287	C	14	dxz	Ryd(4d)	0.00012	2.98888
288	C	14	dyz	Ryd(3d)	0.00042	1.82525
289	C	14	dyz	Ryd(4d)	0.00022	2.84354
290	C	14	dx2y2	Ryd(3d)	0.00082	2.45311
291	C	14	dx2y2	Ryd(4d)	0.00036	3.60667
292	C	14	dz2	Ryd(3d)	0.00109	2.19609
293	C	14	dz2	Ryd(4d)	0.00025	3.28938
300	N	16	S	Cor(1S)	1.99943	-14.47317
301	N	16	S	Val(2S)	1.39728	-0.40799
302	N	16	S	Ryd(4S)	0.01707	1.49930
303	N	16	S	Ryd(3S)	0.00038	1.08834
304	N	16	S	Ryd(5S)	0.00000	35.39732
305	N	16	px	Val(2p)	1.61501	-0.09125
306	N	16	px	Ryd(4p)	0.00867	1.00117
307	N	16	px	Ryd(3p)	0.00045	0.78597
308	N	16	px	Ryd(5p)	0.00009	4.46133
309	N	16	py	Val(2p)	0.98289	0.02984
310	N	16	py	Ryd(4p)	0.01365	1.07899
311	N	16	py	Ryd(3p)	0.00160	0.75086
312	N	16	py	Ryd(5p)	0.00012	4.90360
313	N	16	pz	Val(2p)	1.00556	-0.01496
314	N	16	pz	Ryd(4p)	0.00028	0.81832
315	N	16	pz	Ryd(3p)	0.00017	0.65785

316	N	16	pz	Ryd(5p)	0.00002	4.05992
317	N	16	dxy	Ryd(3d)	0.00568	2.25703
318	N	16	dxy	Ryd(4d)	0.00011	5.23629
319	N	16	dxz	Ryd(3d)	0.00324	1.62841
320	N	16	dxz	Ryd(4d)	0.00003	4.83991
321	N	16	dyz	Ryd(3d)	0.00214	1.68757
322	N	16	dyz	Ryd(4d)	0.00005	4.89550
323	N	16	dx2y2	Ryd(3d)	0.00327	1.92797
324	N	16	dx2y2	Ryd(4d)	0.00028	5.16461
325	N	16	dz2	Ryd(3d)	0.00331	1.88476
326	N	16	dz2	Ryd(4d)	0.00003	5.00706

327	O	17	S	Cor(1S)	1.99985	-19.24327
328	O	17	S	Val(2S)	1.75899	-0.77893
329	O	17	S	Ryd(3S)	0.00800	1.14819
330	O	17	S	Ryd(4S)	0.00027	1.89363
331	O	17	S	Ryd(5S)	0.00000	49.85996
332	O	17	px	Val(2p)	1.85373	-0.15207
333	O	17	px	Ryd(3p)	0.00431	0.94000
334	O	17	px	Ryd(4p)	0.00070	1.32363
335	O	17	px	Ryd(5p)	0.00002	5.40781
336	O	17	py	Val(2p)	1.42646	-0.09848
337	O	17	py	Ryd(3p)	0.00468	0.86741
338	O	17	py	Ryd(4p)	0.00014	1.10333
339	O	17	py	Ryd(5p)	0.00005	5.46642
340	O	17	pz	Val(2p)	1.53569	-0.11073
341	O	17	pz	Ryd(3p)	0.00535	0.65018
342	O	17	pz	Ryd(4p)	0.00037	1.21267
343	O	17	pz	Ryd(5p)	0.00002	5.22447
344	O	17	dxy	Ryd(3d)	0.00095	2.86776
345	O	17	dxy	Ryd(4d)	0.00003	6.88415
346	O	17	dxz	Ryd(3d)	0.00015	2.58735
347	O	17	dxz	Ryd(4d)	0.00000	6.33266
348	O	17	dyz	Ryd(3d)	0.00133	2.69423
349	O	17	dyz	Ryd(4d)	0.00003	6.63320
350	O	17	dx2y2	Ryd(3d)	0.00077	2.87408
351	O	17	dx2y2	Ryd(4d)	0.00005	6.92107
352	O	17	dz2	Ryd(3d)	0.00028	2.72100
353	O	17	dz2	Ryd(4d)	0.00002	6.64220

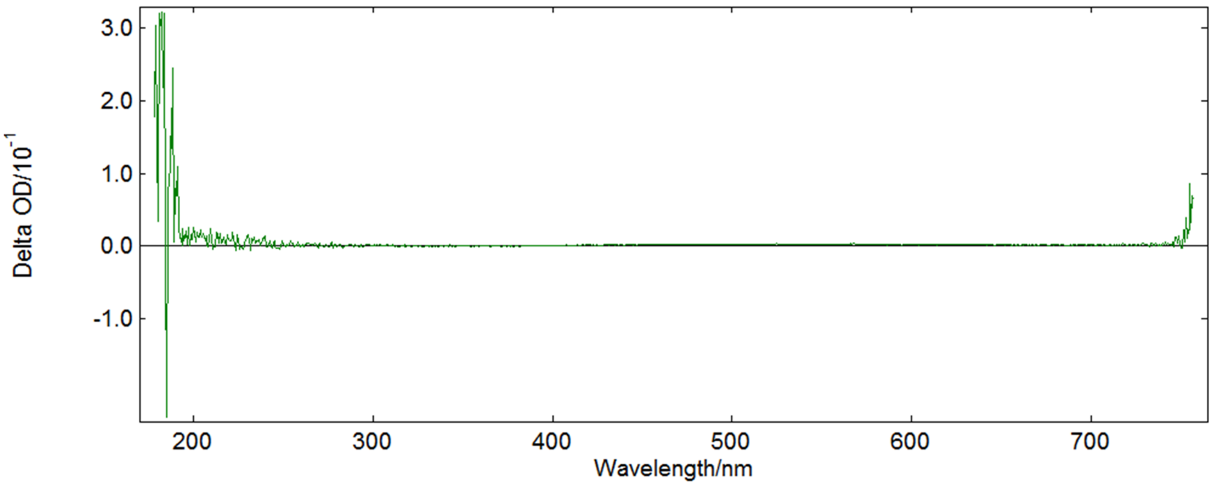

Fig. S12. Transient absorption spectrum of 7-dimethylaminocoumarin oxime **1**/F^- system in MeCN (Edinburgh Instruments Flash photolysis LP980-Spectrometer – nanosecond flash photolysis system; λ_{EX} = 355 nm; T = 298.15 K). Kinetic analysis of the transient absorption spectrum at different wavelength in the region of 200-700 nm does not confirm the presence of any transient decay above 5ns.

Table S4. Excitation energies, oscillator strengths (f) and orbital contributions to corresponding electronic transitions from ground state (S_0) of studied coumarin oxime **1** (geometry was optimized at the M06-2X/6-31+G(d,p) level of theory and energies were calculated at the M06-2X/6-311+G(2d,p) level of theory).

**7-dimethylaminocoumarin oxime 1**					
Excited state	Orbital contributions		Energy		Oscillator strength
			[eV]	[nm]	
T_1	HOMO-2 → LUMO<br>HOMO-1 → LUMO<br>HOMO → LUMO	(6%)<br>(3%)<br>(86%)	2.27	545	0.0000
S_1	HOMO → LUMO	(96%)	3.58	346	0.7375

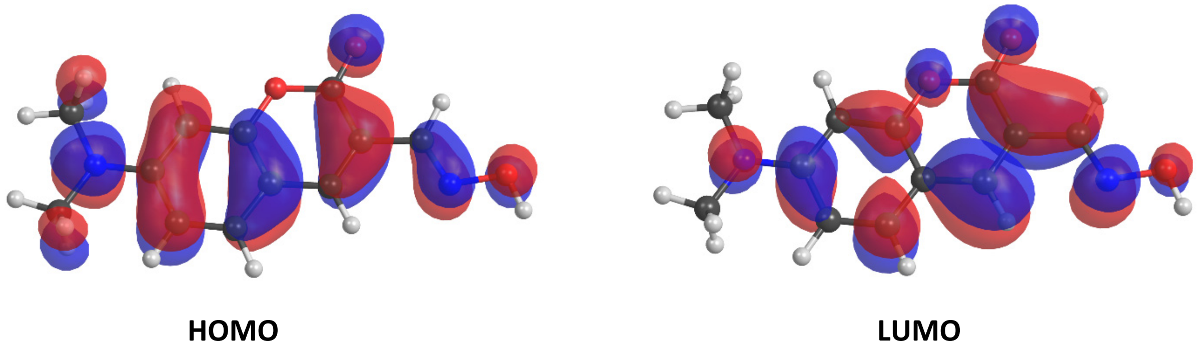

Fig. S13. Frontier molecular orbitals of 7-dimethylaminocoumarin oxime **1** (geometry was optimized at the M06-2X/6-31+G(d,p) level of theory and energies were calculated at the M06-2X/6-311+G(2d,p) level of theory).

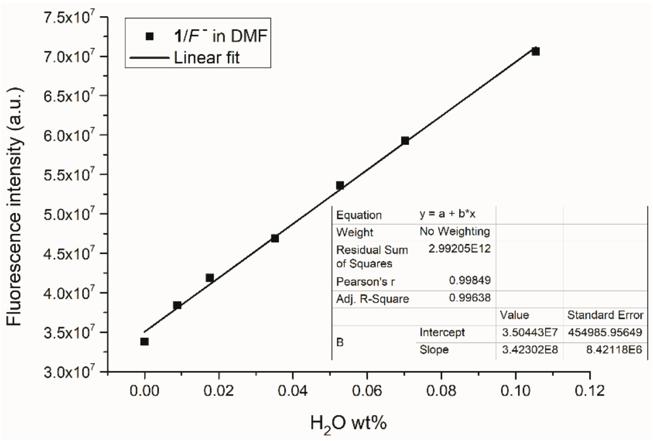

Fig. S14. Fluorescence intensity behaviour of studied two-component coumarin oxime sensor $\mathbf{1}/F^-$ in DMF during titration with water ($\lambda_{\mathrm{EX}} = \lambda_{\mathrm{A(oxime)}}$; $T = 298.15$ K).

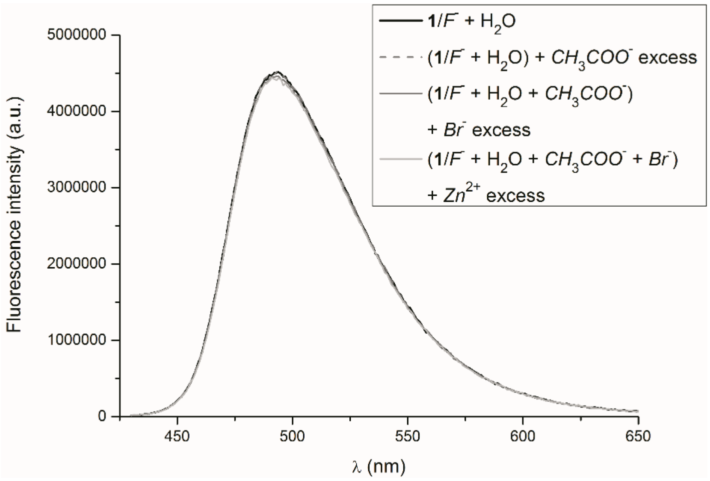

Fig. S15. Fluorescence spectrum of 7-dimethylaminocoumarin oxime $\mathbf{1}/F^-$ system in MeCN after 4 wt% water addition in the presence of various ion excess (~5×10^{-5} mol L^{-1} oxime + 1×10^{-2} mol L^{-1} TBA^+F^-; $\lambda_{\mathrm{EX}} = \lambda_{\mathrm{A(oxime)}}$; $T = 298.15$ K).

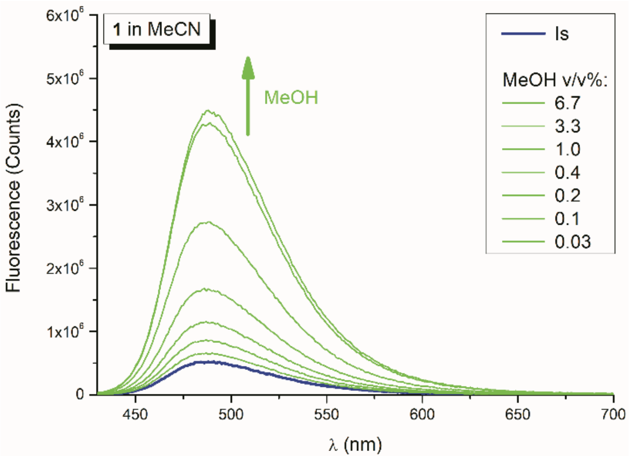

Fig. S16. Evolution of the emission spectrum of 7-dimethylaminocoumarin oxime $\mathbf{1}/F^-$ system in MeCN during $\mathbf{1}/F^-$ solution titration with methanol ($c_1 = 3\times10^{-5}$ mol L^{-1}; $c_{F^-} = 1\times10^{-2}$ mol L^{-1}; $\lambda_{\mathrm{EX}} = 416$ nm; Is – initial state; $T = 298.15$ K).

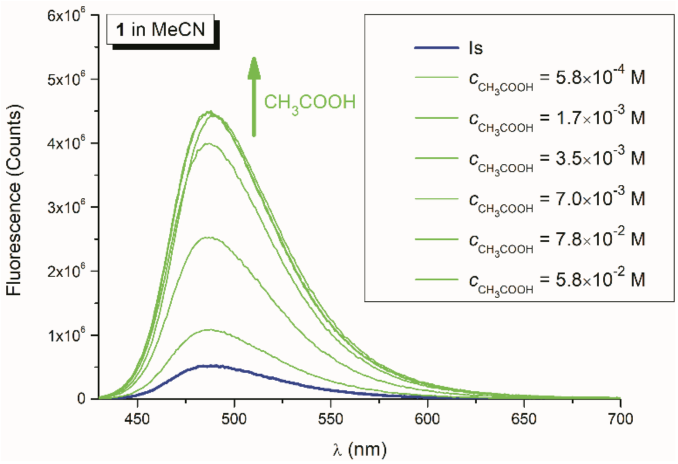

Fig. S17. Evolution of the emission spectrum of 7-dimethylaminocoumarin oxime **1**/F^- system in MeCN during **1**/F^- solution titration with acetic acid ($c_1 = 3\times10^{-5}$ mol L^{-1}; $c_{F^-} = 1\times10^{-2}$ mol L^{-1}; λ_{EX} = 416 nm; Is – initial state; T = 298.15 K; M = mol dm^{-3} = mol L^{-1}).

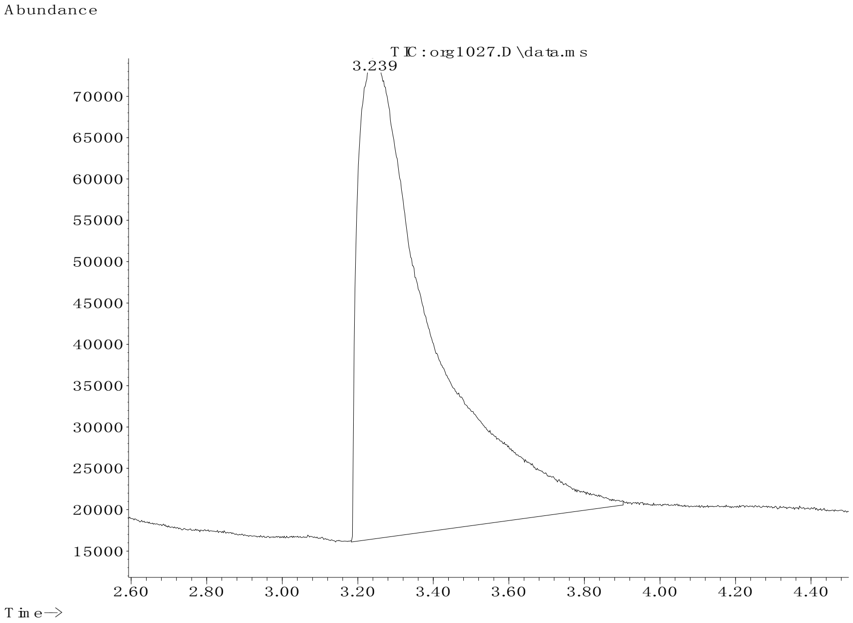

Fig. S18. GC-MS chromatogram of water analysis in MeCN in ionic liquid capillary column (0.4 v/v% of water; x-axis: time in seconds; capillary column coated with a 0.2 µm film thickness of ionic liquid SPB-IL100 as stationary phase was used).

Fig. S19. Coumarin oxime **1:** ^{1}H NMR, ^{13}C NMR.

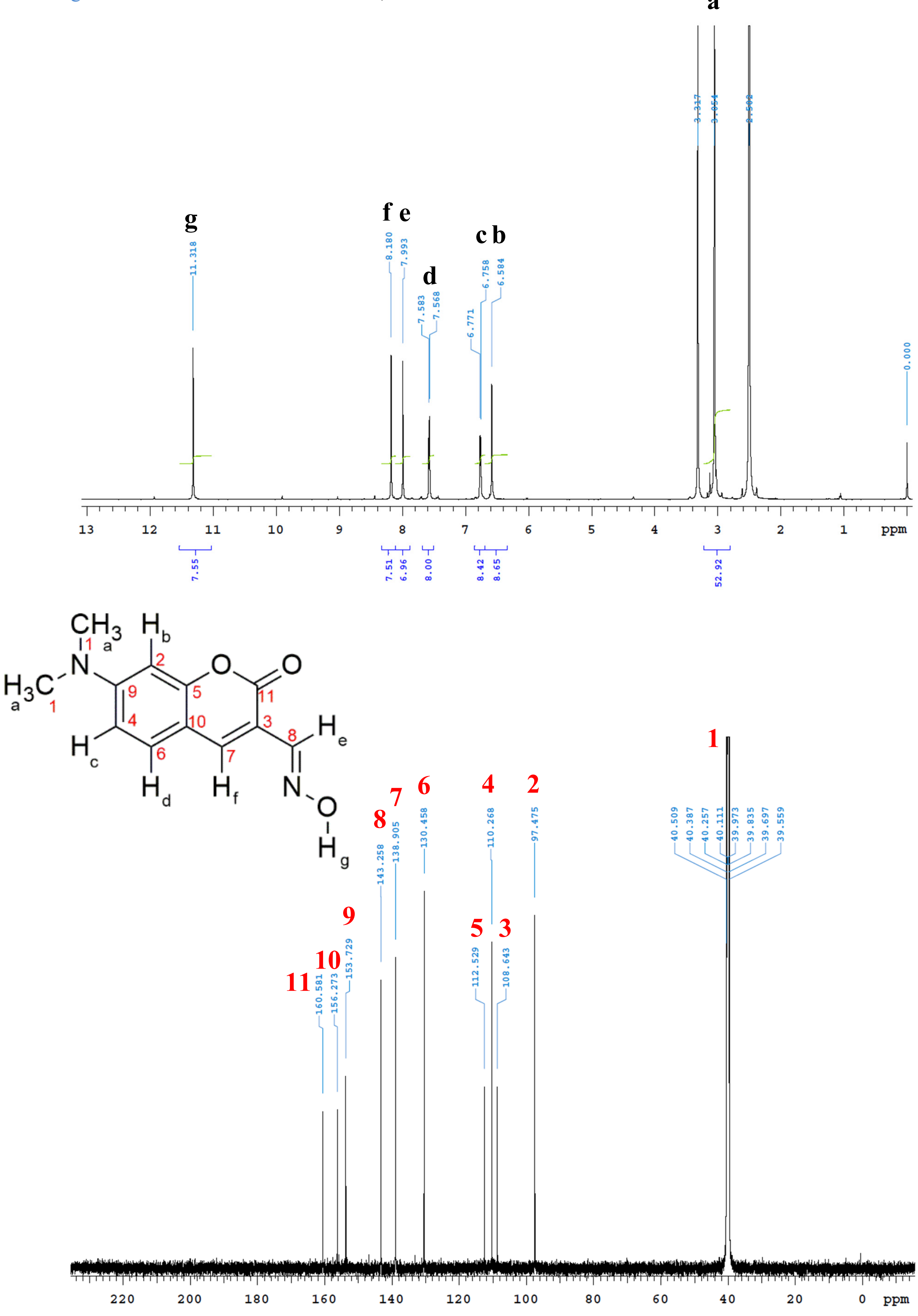

Fig. S19. Coumarin oxime **1:** 2D NMR spectra: HSQC (Heteronuclear Single Quantum Coherence) and HMBC (Heteronuclear Multiple Bond Correlation).

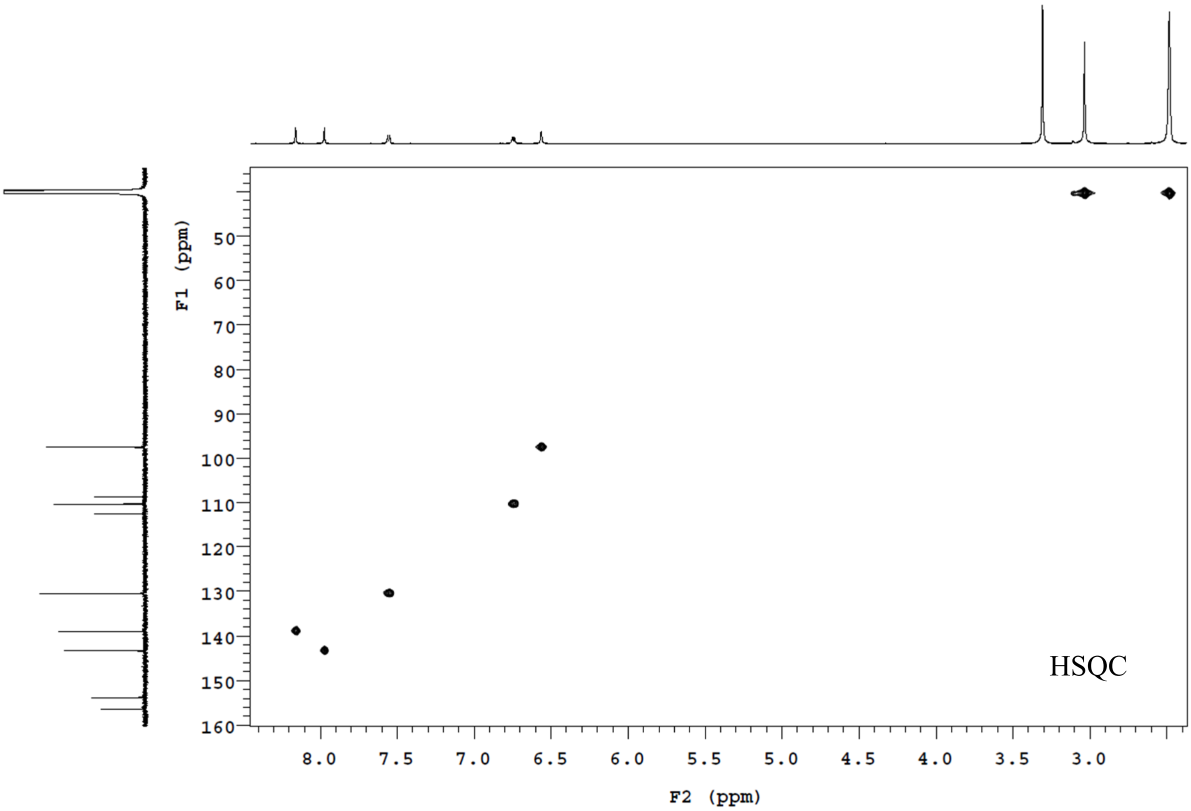

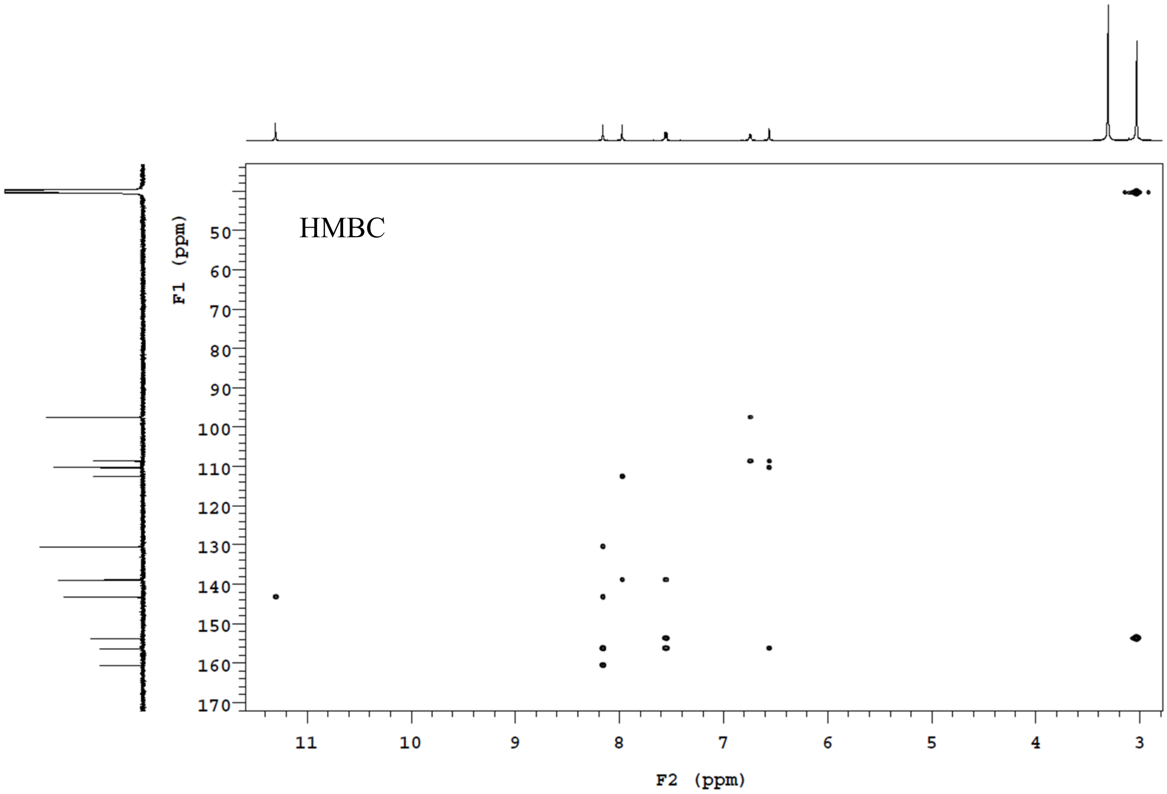

Fig. S20. Coumarin oxime **2:** ^{1}H NMR, ^{13}C NMR.

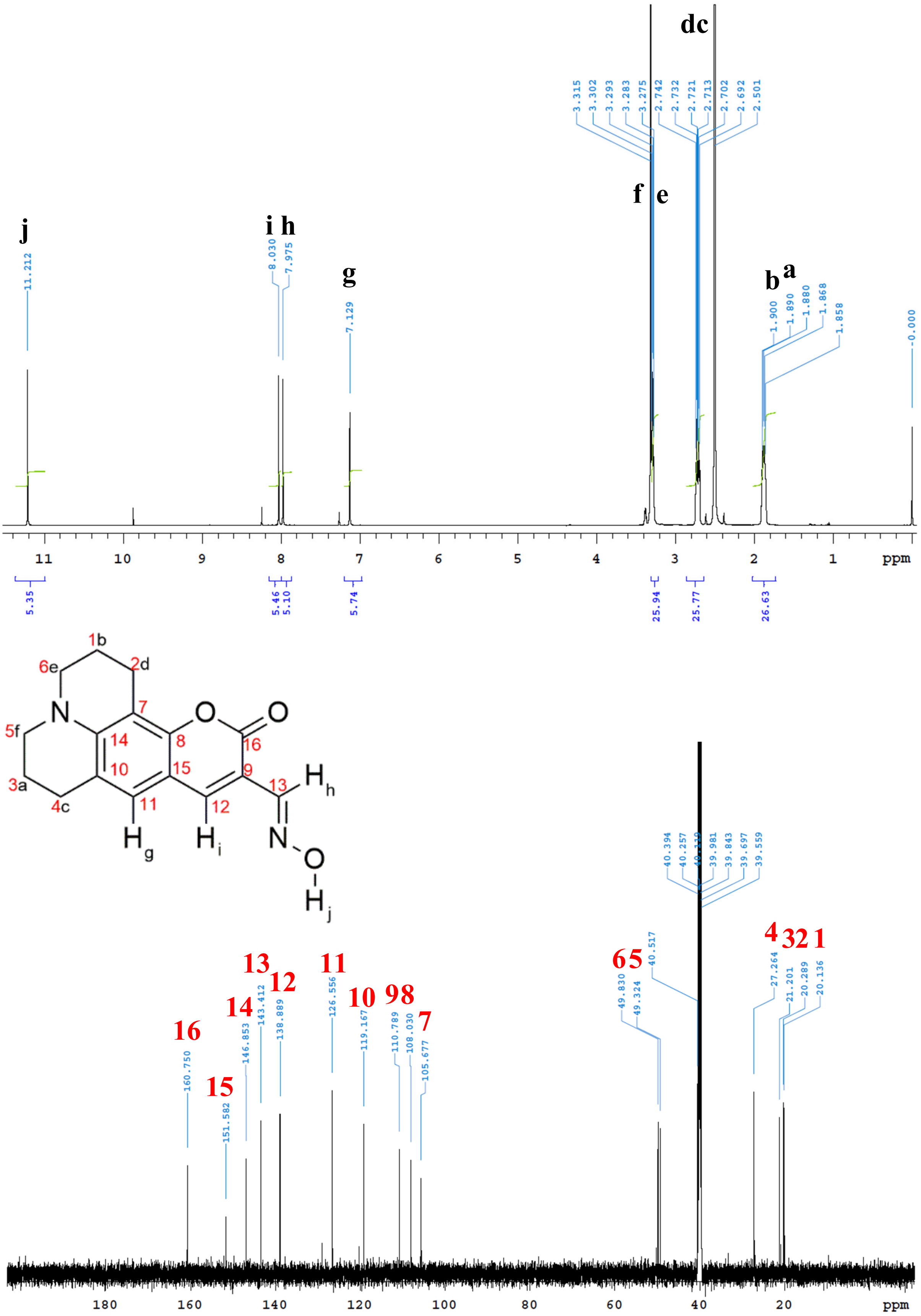

Fig. S20. Coumarin oxime **2:** 2D NMR spectra: HSQC (Heteronuclear Single Quantum Coherence) and HMBC (Heteronuclear Multiple Bond Correlation).

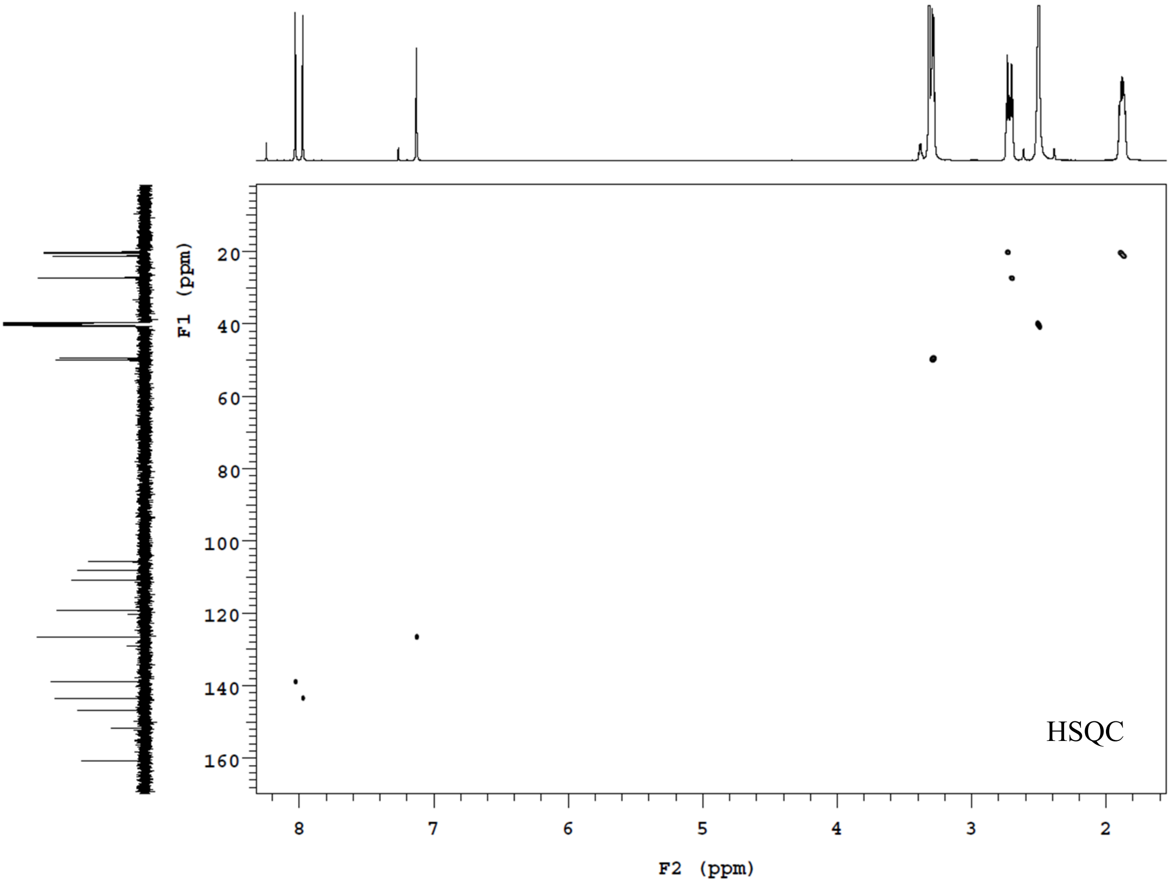

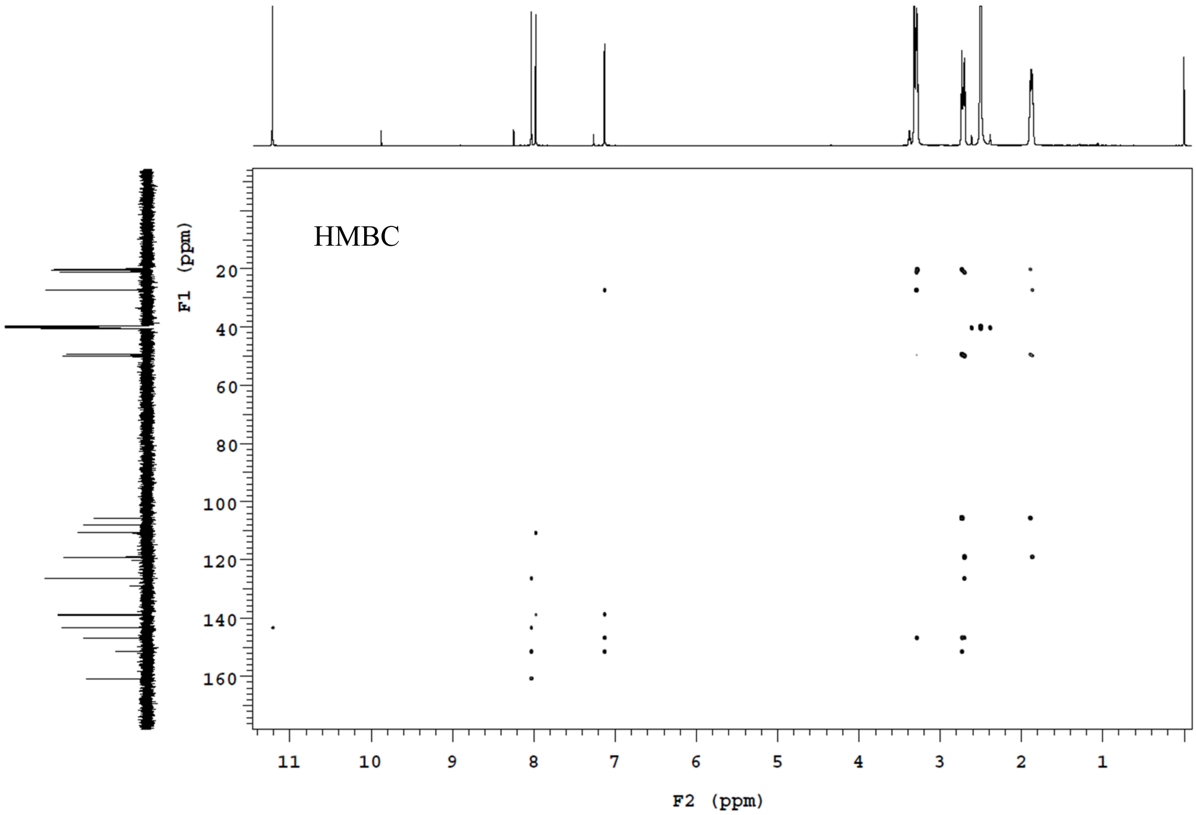

Supplement: Supplementary file 1 [file molecules-22-01340-s001.pdf]
